# Progress in gene editing tools, implications and success in plants: a review

**DOI:** 10.3389/fgeed.2023.1272678

**Published:** 2023-12-07

**Authors:** Suman Jyoti Bhuyan, Manoj Kumar, Pandurang Ramrao Devde, Avinash Chandra Rai, Amit Kumar Mishra, Prashant Kumar Singh, Kadambot H. M. Siddique

**Affiliations:** ^1^ Department of Biotechnology, Mizoram University (A Central University), Pachhunga University College Campus, Aizawl, Mizoram, India; ^2^ Institute of Plant Sciences, Agricultural Research Organization, Volcani Center, Rishon LeZion, Israel; ^3^ Department of Botany, Mizoram University, Aizawl, India; ^4^ The UWA Institute of Agriculture, The University of Western Australia, Perth, WA, Australia

**Keywords:** gene editing tools, zinc finger nuclease, TALEN, CRISPR-Cas, CRASPASE, base editors, prime editors, crops against various environmental challenges including drought

## Abstract

Genetic modifications are made through diverse mutagenesis techniques for crop improvement programs. Among these mutagenesis tools, the traditional methods involve chemical and radiation-induced mutagenesis, resulting in off-target and unintended mutations in the genome. However, recent advances have introduced site-directed nucleases (SDNs) for gene editing, significantly reducing off-target changes in the genome compared to induced mutagenesis and naturally occurring mutations in breeding populations. SDNs have revolutionized genetic engineering, enabling precise gene editing in recent decades. One widely used method, homology-directed repair (HDR), has been effective for accurate base substitution and gene alterations in some plant species. However, its application has been limited due to the inefficiency of HDR in plant cells and the prevalence of the error-prone repair pathway known as non-homologous end joining (NHEJ). The discovery of CRISPR-Cas has been a game-changer in this field. This system induces mutations by creating double-strand breaks (DSBs) in the genome and repairing them through associated repair pathways like NHEJ. As a result, the CRISPR-Cas system has been extensively used to transform plants for gene function analysis and to enhance desirable traits. Researchers have made significant progress in genetic engineering in recent years, particularly in understanding the CRISPR-Cas mechanism. This has led to various CRISPR-Cas variants, including CRISPR-Cas13, CRISPR interference, CRISPR activation, base editors, primes editors, and CRASPASE, a new CRISPR-Cas system for genetic engineering that cleaves proteins. Moreover, gene editing technologies like the prime editor and base editor approaches offer excellent opportunities for plant genome engineering. These cutting-edge tools have opened up new avenues for rapidly manipulating plant genomes. This review article provides a comprehensive overview of the current state of plant genetic engineering, focusing on recently developed tools for gene alteration and their potential applications in plant research.

## 1 Introduction

Recent advancements in gene editing tools and high throughput analysis such as DNA sequencing, the field of genetics is experiencing a golden era. Today, we have numerous ways to conduct whole-genome queries, encompassing sequencing, gene expression, and chromatin status analyses. Over the past few years, we have achieved genome modifications with remarkable precision and specificity (Cong et al., 2013; Baltes et al., 2014; Carroll, 2014; Silva-Pinheiro and Minczuk, 2022).

Historically, genetic studies primarily focused on identifying and examining spontaneous mutations, reflecting the principles laid down by Mendel, Avery, and Morgan, among others (Mendel, 1901; Halligan and Keightley, 2009). In the twentieth century, Auerbach et al. (1947) and Muller (1927) demonstrated that the rate of mutagenesis could be enhanced with chemical or radiation treatment. Later on, techniques using transposon insertions became feasible in some organisms. However, these methods, such as chemical and physical mutagenesis, resulted in modifications to the genome at random sites. In the 1970s and 1980s, the first targeted genetic alterations were achieved in yeast and mice (Scherer and Davis, 1979; Rothstein, 1983; Smithies et al., 1985; Thomas et al., 1986). The gene targeting method relied on homologous recombination, which was precise but inefficient, particularly in mouse cells (Mansour et al., 1988). Applying gene targeting to other species was challenging due to the limited availability of culturable embryonic stem cells in mammals. This limitation was addressed in later advanced gene editing technologies, enabling targeted genetic manipulations in nearly all cell and organism types (Gaj et al., 2013; Carroll, 2014).

Among these advanced techniques, zinc finger nucleases (ZFNs) and transcription activator-like effector nucleases (TALENs) are site-directed nuclease (SDN) facilitated gene editing techniques used in plant genome engineering. The first zinc finger protein was discovered in 1983, marking a significant milestone in molecular biology and genetics (Miller et al., 1985). After discovering ZFNs, later on scientists extensively studied TALEN effectors and clustered regularly interspaced short palindromic repeats (CRISPR) (Ishino et al., 1987; Boch et al., 2009). The fundamental idea behind the ZFN system was to genetically modify nucleases to create DNA double-strand breaks (DSBs) at specific sites. This concept opened up new avenues of research, ranging from applied biotechnology to fundamental biomedical science (Ran et al., 2018; Xu et al., 2020; Goswami et al., 2022). The subsequent development of ZFNs and TALENs involved fusing zinc fingers and transcription activator-like effectors with the Fok1 DNA cleavage domain. Fok1, being a dimer, requires the precise molecular design of ZFNs and TALENs to bring the two domains of Fok1 together for catalytic activity (Bak et al., 2018). However, designing and constructing ZFNs in plants posed challenges due to their expense and susceptibility to inaccurate DNA sequence recognition (Miller et al., 2007). TALENs offered improved target binding specificity, but were complex to synthesize due to the repetitive nature of DNA-binding domains in the TALEN protein, leading to lesser precision and reduced target-cutting efficiency (Gaj et al., 2013). Consequently, TALENs and ZFNs faced limitations in their widespread utilization.

The discovery of CRISPR-Cas revolutionized gene editing and quickly became the most advanced and well-known platform for nucleases in gene editing (Cong et al., 2013). Originally a defense mechanism providing immunity to bacteria against bacteriophages, the CRISPR-Cas system uses a single guide RNA (sgRNA), composed of nearly 100 nucleotides, that directs the Cas enzyme to the target site on the DNA, leading to DSBs (Westra et al., 2012). The simplicity, specificity in action, cost-effectiveness, and rapidity of CRISPR-Cas have contributed to its immense popularity (Rahim et al., 2021). Its high-efficiency gene editing capability, inducing desired chromosomal sequences from targeted DNA through DSBs, has solidified its position as a leading gene editing tool. CRISPR-Cas tool have been successfully used for precise modifications of genes related to abiotic stress responses and improved the crop performance against various environmental challenges including drought, salinity, extreme temperature, heavy metal stress (Shan et al., 2013; Shi et al., 2017; Xu et al., 2015; Zhao et al., 2016). In biotic stresses CRSIPR used for editing of genes related to disease resistance caused by bacteria, viruses and fungi, also for insect resistance. This can reduce the need for chemical pesticides, making agriculture more sustainable (Ali et al., 2015; Wang et al., 2016; Zhang et al., 2022; Zhou et al., 2015). CRISPR for RNA editing is become a powerful technique that complements traditional DNA editing methods, offering flexibility and precision in tailoring crop traits for improved agricultural outcomes (Mao et al., 2018). CRISPR tool has been successfully employed for metabolic engineering which allows precise editing of genes involved in particular metabolic pathway for nutrient enrichment, crop quality improvement (Selma et al., 2023). Recently, Wang and Doudna. (2023) reviewed advancement in the CRISPR gene editing technology, successes and their limitations, and highlighted some examples of how CRISPR is being used in medicine and agriculture and exciting future opportunities. There are some reviews recently published, which mainly focused on the advancement in gene editing of plants, animals and other organisms (Anzalone et al., 2020; Laforest and Nadakuduti, 2022; Sprink et al., 2022; Yang et al., 2021).

A new member, CRASPASE, a CRISPR RNA-guided protease, has emerged as a promising alternative to Cas nucleases, as it does not interact with DNA, making it potentially suitable for various therapeutic applications in the future (Hu et al., 2022). In addition, two novel gene editing tools, base editors and prime editors, have been introduced, offering greater efficiency and fewer by products in non-dividing or slowly dividing cells compared to nuclease-dependent methods, which often result in uncontrolled editing outcomes, including chromosomal rearrangements, larger deletions, and combinations of insertions and deletions (indels). In the ensuing section, we put forward a brief description about different gene editing tools, i.e., zinc finger nucleases, transcription activator-like effector nucleases, (CRISPR)-Cas variants, CRASPASE, base editors and prime editors.

## 2 Zinc finger nucleases (ZFN)

Zinc finger nucleases (ZFNs) are one of the oldest tools used for gene editing in various organisms, including plants, fungi, animals, and bacteria. They consist of a synthetic zinc finger-based DNA-binding domain engineered to precisely bind to the target DNA sequence and a Fok1 domain that cleaves the target sequence (Osakabe and Osakabe, 2015). ZFNs can be used to cleave at any genomic sequence by engineering zinc finger DNA-binding domain. The Fok1 nuclease domain, when fused with the zinc finger array, forms dimers from monomers and is responsible for cutting the target DNA site (Osakabe and Osakabe, 2015; Petolino, 2015). The amino acids at the start position of zinc finger α-helix are mainly responsible for site-specific binding to the target DNA sequence and can be manipulated to bind any specific targeted sequence (Durai et al., 2005; Camenisch et al., 2008). Various studies have described and set rules for selecting target DNA sequences for ZFNs (Mandell and Barbas, 2006; Sander et al., 2007). Context-dependent assembly (CoDA) and oligomerized pool engineering (OPEN) are two systems for engineering zinc finger arrays for plant gene modification. CoDA uses pre-selected two-finger units for rapid assembly, while OPEN uses genetic selection to engineer zinc finger arrays (Maeder et al., 2008; Townsend et al., 2009; Zhang et al., 2010; Sander et al., 2011; Curtin et al., 2012). Two ZFNs are required to bind opposite DNA cleavage sequences separated by 5–7 bp, and dimerization of the fok1 nuclease domain is required to cut the target DNA site when ZF-binding sites are palindromic (Bae et al., 2003; Segal et al., 2003; Shukla et al., 2009). Fok1 dimerizes and creates a DSB in the spacer region between the two opposite strands. The DSB is then repaired by natural repair systems such as homologous recombination (HR) and non-homologous end joining (NHEJ), causing a site-specific mutation in that sequence, with NHEJ resulting in gene disruption and HR integrating exogenous sequences (Urnov et al., 2005; Christian et al., 2010).

One study showed that ZFN-mediated gene targeting could be used as a transient expression system in tobacco, with a gene targeting efficiency of 4% (Townsend et al., 2009). In another study, ZFNs were successfully integrated into targeting gene *IPK1* at a specific site in the maize genome to increase gene targeting frequency by introducing heterologous donor DNA molecules into maize cells. *IPK1* gene in maize encodes an enzyme known as inositol- 1,3,4,5,6-pentakisphosphate 2-kinase responsible for catalyzing the final step of phytate synthesis (Shukla et al., 2009). Moreover, 20% of the selected lines exhibited inheritable gene targeting events inherited into the next-generation. These findings demonstrate that the targeted cleavage of the genome by ZFNs significantly enhances HR-mediated gene targeting in plants. ZFNs are also used as a targeted mutagenesis tool for plant genome editing. In Arabidopsis, Lloyd et al. (2005) reported target-specific mutations using a heat shock promoter (HSP), and Osakabe et al. (2010) studied ZFN-mediated mutagenesis of the *ABSISCIC ACID INSENSITIVE 4* gene using an HSP and reported a mutant phenotype similar to an ABA-insensitive mutant studied earlier by Gazzarrini and McCourt (2001), Finkelstein et al. (2002), Finkelstein and Gibson (2002), León and Sheen (2003). Ainley et al. (2013) reported site-specific multiple trait stacking by ZFNs in maize, generating maize plants with additional synthetic ZFN target sites and a herbicide-resistant marker and introducing another herbicide-resistant marker gene using a target site-specific ZFN and synthetic ZFN target site. In Arabidopsis, alcohol dehydrogenase and chalcone synthase genes have been mutated using site-directed mutagenesis with ZFNs driven by estrogen-inducible system (Zhang et al., 2010). These studies, among others, have used ZFNs to create DSBs and induce NHEJ repair to produce mutant plants (Tovkach et al., 2009, 2010). In soybean, the ZFN mutagenesis tool was used to cause mutation in two paralogous *DCL4b* and *DCL4a* DICER-LIKE (*DCL*) genes with estrogen-inducible promoter, resulting in an adenine base insertion and two base adenine and thymine insertions, respectively, with the mutant plant exhibiting phenotypic abnormalities such as aborted seeds (Curtin et al., 2011). This tool has been used for the deletion of large sequences of genomic loci; for instance, in tobacco plants, ZFN-mediated cleavage deleted 2.8 kb of the targeted sequence (Cai et al., 2009). In another study, ZFN-mediated cleavage deleted 4.3 kb of integrated *GUS* gene sequence (Petolino et al., 2010), with an even larger deletion of 55 kb reported after nuclease cleavage within tandem gene clusters (Voytas, 2013). These reports suggest that ZFN is a powerful tool for targeted gene sequence deletions in plants. In *Arabidopsis*, ZFN-mediated DSB repaired by homology-directed repair (HDR) edited the PROTOPORPHYRINOGEN OXIDASE (*PPOX*) gene, resulting in butafenacil herbicide resistance (de Pater et al., 2013). In perennial fruit trees such as apple and fig trees, ZFN-mediated gene modifications resulted in precise gene editing (Peer et al., 2015). In another study, custom designed ZFNs for LEAFY-COTYLEDON1-LIKE4 (*L1L4*) gene, when transiently expressed in tomato seeds, successfully cleaved the target site and induced mutations in *L1L4* gene, revealing hetero-chronic phenotypes during developmental stages (Hilioti et al., 2016). *GUS:NPTII* reporter genes integrated into different chromosomal sites in ten tobacco lines showed high-frequency HR using a ZFN gene targeting system (Wright et al., 2005). In another study, precise modifications to a single amino acid in the *acetohydroxyacid synthase* gene of allohexaploid bread wheat (*Triticum aestivum*) using ZFN and a DNA repair template resulted in imidazolinone herbicide resistance, a stable trait that was transmitted to next-generation. Schiermeyer et al. (2019) recently developed an engineered transgene integration platform (ETIP) for the successful integration of donor DNA sequences thorough NHEJ or HDR mechanisms, with ZFNs facilitating the donor DNA integration in *Nicotiana tabacum* BY-2 cells, the targeted DNA integration success rate was achieved 41% for NHEJ and upto 21% for HDR. At the insertion site, ETIP mainly uses incomplete marker genes, the two marker genes, encoding *Discosoma* sp. red fluorescent protein (*DsRed*) and neomycin phosphotransferase II (*nptII*) which enable event selection on kanamycin containing selective medium and screening for red fluorescent clones. The ETIP cassette transformed into *Nicotiana tabacum* BY-2 suspension cells. The fundamental problem with ZFNs is that their specificities might overlap and depend on the context of the DNA and zinc fingers around them. The zinc finger array must be built for each editing, and there is only a finite amount of good targeting sites. The limitations of ZFNs include complex, expensive, and technically challenging modules with certain difficulties in replacing larger fragments in the creation of knockouts. Furthermore, because there are only a limited number of target sites available, ZFN application can occasionally go off-target as a result of non-specific DNA binding (DeFrancesco, 2011). The use of ZFNs in genome editing has always been difficult and has not been extensively adopted by the research community, despite the existence of multiple successful studies.

## 3 Transcription activator-like effector nucleases (TALENs)

The emergence of TALEN technology, another protein-based DNA-targeting tool, has transformed applied biology due to its improved sequence precision and reduced cytotoxicity compared to other DNA-binding proteins like ZFNs (Joung and Sander, 2013). TALENs are more mutagenic than ZFNs (Becker and Boch, 2021). TALENs, as a second-generation gene editing tool, comprise the fok1 nuclease domain and Transcription Activator-Like Effector (TALE) protein, containing customizable DNA-binding repeats. TALE proteins are DNA-specific binding proteins produced by pathogenic bacteria belonging to the *Xanthomonas* genus, which infect plants. TALE proteins consist of alpha-helical hairpin domains, the basic building blocks. They primarily regulate host gene expression by binding with specific regions in the promoter sequence of the host gene in the nucleus and initiate transcription (Wright et al., 2014).

Each TALE repeat comprises 33–35 amino acid residues and recognizes one DNA nucleotide using a highly variable residue within the repeat at a fixed position. The specific arrangement of these repeat variable di-residues (RVDs) (Becker and Boch, 2021) generates a one-repeat-to-one base pair code, allowing a single TALE to recognize a specific long DNA sequence (Sprink et al., 2015; Malzahn et al., 2017).

Several modular assembly strategies, such as the golden gate cloning method, PCR-dependent or ligation-independent sub-cloning, and a high-throughput automated solid phase method, have been developed to construct TALENs (Becker and Boch, 2021). Recently, using mitochondria-targeting TALEN-based cytidine deaminase successfully substituted C:G pairs with T:A in mitochondrial genome of *Arabidopsis thaliana* without causing any changes in genome structure and stably inherited into next-generation and made it possible to carry out targeted base editing of mitochondrial genome (Nakazato et al., 2022). For chloroplast and mitochondrial gene editing Kang et al. (2021) developed golden gate cloning system to deliver 16 expression plasmids (8 in chloroplast and 8 in mitochondria) and delivered DddA-derived cytosine base editor (DdCBE) plasmids to cause point mutation in both the organelles in lettuce and rapeseed calli and induced base editing with frequency upto 25% in mitochondria and 35% in chloroplasts. The TALEN approach can target each organelle by linking to the sequences of a mitochondrial targeting signal or plastid target peptide (Arimura, 2022). However, inducing DSBs in the mitochondrial genome can trigger large deletions that alter the genome structure. The discovery of advanced gene editing methods, such as prime editors and base editors have enabled precise gene editing without adverse effects. While TALENs offer advantages over ZFNs with their repetitive variable double amino acid residues that can detect a variety of bases, they present technical challenges and can be more expensive and less efficient when implemented for gene editing. When compared to ZFNs, the number of potential target sites for TALENs is substantially greater because any nucleotide base could be the target of a TALE repeat. The choice of the desired target sequence turns out to be constrained because the thymidine nucleotide at the 5′ end of the target DNA influences the half-repeat’s ability to attach. However, this restriction can potentially be removed by choosing mutant TALEN N-terminal domain variants that can bind to A, G, or C (Lamb et al., 2013).

## 4 Clustered regularly interspaced short palindromic repeats (CRISPR)-Cas

**FIGURE 1:**
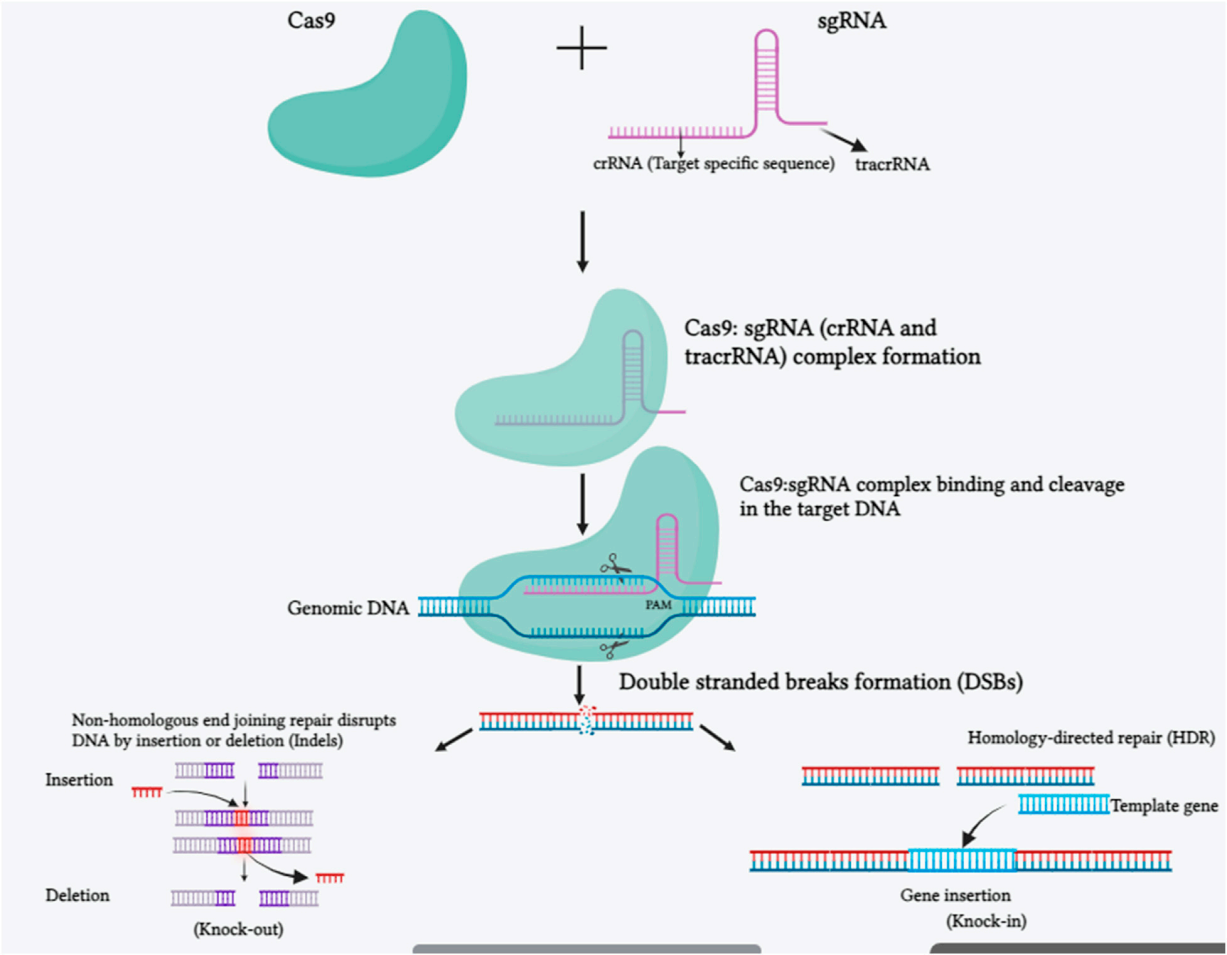
The molecular mechanism of the CRISPR-Cas9 system is depicted in a schematic picture. SgRNA and Cas9 make up the CRISPR/Cas 9 system. The trans-activating crispr RNA (tracrRNA) and crispr RNA (crRNA) are components of the sgRNA. The crRNA is made up of a 20 nucleotide protospacer element and a few more nucleotides that are complementary to the tracrRNA. In order to edit the genomic sequences, the tracrRNA hybridizes with the crRNA and attaches to the Cas9 protein, making the CRISPR-Cas9/sgRNA complex. The Cas9-sgRNA complex unwinds the dsDNA, and the sgRNA complementary sequence anneals to one of the DNA strands. The endonuclease domains of the Cas9 protein break both DNA strands three bases upstream of the protospacer-adjacent motif (PAM) region after binding. Once a double-strand break (DSB) occurs in DNA, it is repaired either by a homology-directed repair (HDR) route or by non-homologous end joining (NHEJ). HDR repair enables for precise genome editing at the target region, whereas NHEJ causes small insertions, deletions, or indels (Created with BiorRender.com).

Clustered Regularly Interspaced Short Palindromic Repeats (CRISPR)/CRISPR-associated system (Cas) is the third-generation gene editing tool that has revolutionized biotechnology research. CRISPR was first discovered in *E. coli* in 1987 and subsequently found in many other bacteria (Garneau et al., 2010). However, its role remained unclear until studies in 2005 revealed its involvement in RNA-mediated DNA cleavage and adaptive immune responses against potentially harmful foreign DNA (Haft et al., 2005). CRISPR-Cas technology harnesses two essential elements—single-stranded guided RNA (sgRNA) and the Cas enzyme—to manipulate the genomes of various organisms. **Single-stranded guide RNA (sgRNA)** is composed of two parts: CRISPR RNA (crRNA) which is 17–20 nucleotide sequence complementary to the target sequence and trans-activating crRNA (tracrRNA) scaffold mainly guides the Cas9 nuclease to the specific nucleotide sequence of the target DNA (Gaj et al., 2013). Binding of crRNA and tracrRNA forms the functional sgRNA; a longer RNA scaffold binds to the DNA target mainly by crRNA portion of the sgRNA, and the Cas9 nuclease is directed to the specific nucleotide region by sgRNA. The crRNA sequence of the sgRNA can be engineered to complement the target DNA sequence, allowing it to bind to the specific region of the genome (Lino et al., 2018). **Cas enzyme** functions as molecular scissors, cutting the target DNA sequence at specific sites guided by the sgRNA, thus enabling small DNA fragments to be added or removed (Figure 1). Theoretically, the sgRNA should not bind to any other sequence in the genome except the target sequence. The delivery tools for plant transformation of CRISPR-Cas construct is mainly agrobacterium mediated transformation, here T-DNA will be incorporated in plant genome. Particle bombardment method using gene gun is mostly used for monocot. Protoplast transfection with recombinant plasmid DNA expressing gene-editing reagents and regeneration of whole plants from these transformed protoplast cells is achieved in many crop species (Woo et al., 2015; Andersson et al., 2018; González et al., 2020). Another method for heritable gene editing using tobacco rattle virus (TRV) contains positive strand RNA, used to deliver sgRNA in cas9-overexpressing plants through agrobacterium infiltration (Ellison et al., 2020). Mobile guide RNAs and nanoparticles are also used for CRISPR-Cas construct delivery in plant gene editing (Demirer et al., 2019; Nadakuduti and Enciso-Rodríguez, 2021). Recent studies of successes in the improvement of crops using CRISPR-Cas is summarized in Table 1.

**TABLE 1:** List of recent successes in the improvement of crops with the use of clustered regularly interspaced short palindromic repeats (CRISPR-Cas).

Associated trait	Target gene/Cas variant	Gene function	Modification efficiency/Result	Cultivar	Transformation method	References
Yield (seed size and seed number)	*BnaEOD3*	Controls seed size, silique length, and seed production in rapeseed	Knockout	*Brassica napus*	*Agrobacterium-*mediated transformation	Khan et al. (2020)
	Cas variant: Cas9		Seed weight increased in the quadruple mutants by 13.9%			
Yield (pod shatter-resistant)	*BnSHP1*	*BnSHP1A09* regulation of lignin composition at the dehiscence zone	Knockout	*Brassica napus*	*Agrobacterium*-mediated transformation	Zaman et al. (2021)
	*BnSHP2*		Pod-shattering resistance index (SRI) increased in mutant lines (0.31) compared to the wild-type (WT) (0.036)			
	Cas variant: Cas9					
Yield (plant architecture)	*BnaBP*	Regulation of pedicel bending and leaf morphogenesis in *Arabidopsis*	Knockout	*Brassica napus*	*Agrobacterium*-mediated transformation	Fan et al. (2021)
	Cas variant: Cas9	Downregulation of *BnaBP* genes decreases the branch angle to create more compact plants	Plant height decreased in the mutant plants by 15.8%–16.9%			
			Branch angle decreased from 84° (WT) to 14° in mutant plants			
Plant yield and architecture	*BnaMAX1*	*bnaMAX1* gene controls axillary bud outgrowth and plant height	Knockout	*Brassica napus*	*Agrobacterium tumefaciens* mediated floral dip method	Zheng et al. (2020)
	Cas variant: Cas9		Plant height decreased by 31.9%–36.5% and total silique number increased by 62.3%–71.8% in mutant plants compared to the WT			
Fatty acid composition and oil content (oil content)	*BnLPAT2*	*BnLPAT2*/*BnLPAT5* plays a major role in regulating the morphology and number of oil bodies	Knockout	*Brassica napus*	*Agrobacterium-*mediated hypocotyl transformation method	Zhang et al. (2019)
	*BnLPAT5*		Mutation frequency: 17%–68%			
	Cas variant: Cas9		No mutation observed in off-target sites			
Fatty acid composition and oil content (oleic acid content)	*BnaFAD2*	*Fatty acid desaturase-2* (*FAD2*) gene impacts fatty acids, mainly oleic, linoleic, and linolenic, in oilseed plants	74.1% short-nucleotide alterations (≤3 bp), with 51.9% single nucleotide insertions and deletions	*Brassica napus*	*Agrobacterium-*mediated hypocotyl transformation method	Huang et al. (2020)
	Cas variant: Cas9					
Isoflavone content	*GmF3H1*, *GmF3H2* and *GmFNSII-1*	Isoflavone synthesis	Knockout	*Glycine max*	*A. rhizogenes*-mediated method and *Agrobacterium*-mediated cot node transformation	Zhang et al. (2020)
	Cas variant: Cas9		Mutation efficiency increased by 44.44%, and triple gene mutation observed in T0 transgenic plants			
Yield (blocking *OsAAP3* increases grain yield by increasing tiller number in rice)	*OsAAP3*	*OsAAP3* gene increases rice tiller number and elongation of outgrowth bud by regulating the concentrations of Lys, His, Ala, Asp, Arg, Gln, Gly, Tyr, and Thr in rice	Knockout and overexpression	*Oryza sativa*	*Agrobacterium*-mediated transformation	Lu et al. (2018)
	Cas variant: Cas9		Tiller numbers increased in *OsAAP3* knockout lines and decreased in *OsAAP3* OE lines compared to WT plants			
Yield (grain weight, grain number, and grain size)	*OsGS3, OsGW2* and *OsGn1a*	Negatively regulate grain weight, width, number, and size	Off-target mutation Mutation frequency: 66.7%–100%	*Oryza sativa*	*Agrobacterium*-mediated transformation	Zhou et al. (2019)
	Cas variant: Cas9	*GN1a* encodes a cytokinin oxidase/dehydrogenase, OsCKX2 that negatively regulates grain size				
Increased resistance to bacterial blight	OsSWEET11 (PthXo1) and OsSWEET14 (PthXo3/AvrXa7)	SWEET genes are susceptibility (S) genes and encode sugar transporter proteins. As a result, recessive alleles of these SWEET genes confer resistance	Knockout	*Oryza sativa*	*Agrobacterium*-mediated transformation	Xu et al. (2019)
	Cas variant: Cas9		Using the CRISPR/Cas9 system, a new germplasm named MS134K was developed with mutated EBE alleles of OsSWEET13, OsSWEET14, and OsSWEET11, providing excellent resistance to Xoo strains			
Cotton genome editing efficiency by Cas 12	Deoxyglucose-5-phosphate synthase (*GhCLA*)	Terpenoid biosynthesis	Knockout	*Gossypium hirsutum*	*Agrobacterium*-mediated genetic transformation	Wang et al. (2020)
	Cas variant: Cas12b (C2c1) from *Alicyclobacillus acidoterrestris* (AacCas12b)		Mutation rate: ∼20%			
Yield (disease resistance)	xopV	XopV suppresses PTI peptidoglycan-triggered response in rice	Knockout	Oryza sativa	Electro-transformation	Yan et al. (2023)
	Cas variant: Cas12a from Francisella novicida		CRISPR-NHEJ method produced high editing frequencies (40.91%–95.45%)			
Plant architecture and fruit ripening	*SlBRI1*, *SlRIN*	*slBRI1* gene performs an essential function in controlling plant architecture	Knockout	*Solanum lycopersicum*	*Agrobacterium*-mediated genetic transformation	Niu et al. (2020)
	Cas variant: XNG-Cas9	*SlRIN* gene has a vital role in the ripening of fruits	XNG-Cas9 edited the AGG site with 13.3% efficiency. Other editing efficiencies: 23.5% at GGC, 22.2% at GGT, and 15.2% at TGA sites., Mutation efficiency of 15.8% at AGA site			
Carotenoid biosynthesis	*PDS3* (phytoene desaturase)	*PDS3* gene positively regulates carotenoid biosynthesis	Knockout	*Arabidopsis thaliana*	Protoplast transfection	Pausch et al. (2020)
	Cas variant: Cas Ф		8–10 bp deletions in PDS3 genes			
Fatty acid composition and oil content (oil content)	Glyma10g42470 *and* FAD2-A	Controls oleic acid content in developing soybean seed	Knockout	*Glycine max*	*Agrobacterium*-mediated soybean hairy root transformation *Agrobacterium rhizogenes*	Duan et al. (2021)
	Cas variant: Cpf1 from Lachnospiraceae *bacterium ND 2006* (LbCpf1)		CRISPR/LbCpf1 induced deletions of huge chromosomal segments with up to 91.7% editing efficiency			
Ureide biosynthesis	XDH, NSH1, NSH2, XMPP *and* GSDA	All genes mentioned are involved in ureide biosynthesis	The onset of hairy root development activates the automatic repair mechanism when Cas9 induces double-strand breaks. Determined the xanthosine and guanosine are key metabolites needed for ureide production	*Phaseolus vulgaris*	R. rhizogenes	Voß et al. (2022)
	Cas variant: Cas9					
Loss of seed and pod development	Vu-SPO11	Mutation in this gene causes fertility loss, with no seed or pod development	Knockout	*Vigna unguiculata*	*Agrobacterium*-mediated genetic transformation)	Che et al. (2021)
	Cas variant: *spCas9*		Effective gene editing			
			Modification frequencies: 4%–37%			
Disease resistance	Two *CsWRKY22* alleles	Decreases Wanjincheng orange susceptibility to *Xanthomonas citri* subsp. Citri	Indels and nucleotide substitutions	*Citrus sinensis*	*Agrobacterium*-mediated genetic transformation	Wang et al. (2019)
			Mutation rates of mutant lines:			
	Cas variant: Cas9		W1-1: 85.7%			
			W2-2: 79.2%			
			W2-3: 68.2%			
Crop improvement	*OsALS, NRT1.1B, OsCDC48and OsWaxy*	OsALS: provides imazamox herbicide resistance in rice	Base edit	*Oryza sativa*	*Agrobacterium*-mediated genetic transformation	Xu et al. (2019)
		*NRT1.1B* gene increases nitrogen use efficiency in rice plants	At the various targets, the editing efficiency increased 2- to 3-fold in the three high-fidelity Cas9 variants			
	Cas variant: Three SpCas9 variants, SpCas9-HF2, HypaCas9 and eSpCas9 (1.1)	*OsCDC48* regulates cell death and senescence				
		*OsWaxy* plays a vital role in granule-bound starch biosynthesis				
Crop improvement (chlorsulfuron-resistant plants)	*SlALS1*	Acetolactate synthase (*ALS*) gene is important for branched-chain amino acid biosynthesis	Base edit	*Solanum tuberosum* and *Solanum lycopersicum*	*Agrobacterium*-mediated genetic transformation	Veillet et al. (2019)
	Cas variant: nCas9 cytidine base editing system		Homologous gene selection and sequencing			
			Modification efficiency: 71%			

In CRISPR-Cas gene editing, off-target effects may not provide a significant barrier in the majority of plant species, but they are likely in paralogs with higher sequence identity, such as those studied in maize, rice, and soya bean (Jacobs et al., 2015; Li et al., 2016; Shan et al., 2013). In contrast to spontaneous and somaclonal mutations, whole genome sequencing studies of Cas9- and Cpf1-edited plants in rice suggested that off-target mutations generated by these enzymes are low (Tang et al., 2018). Therefore, the initial step in improving the specificity of the gene editing might very easily be computational selection of gRNA (Heigwer et al., 2014).

### 4.1 Types of CRISPR-Cas system

**FIGURE 2:**
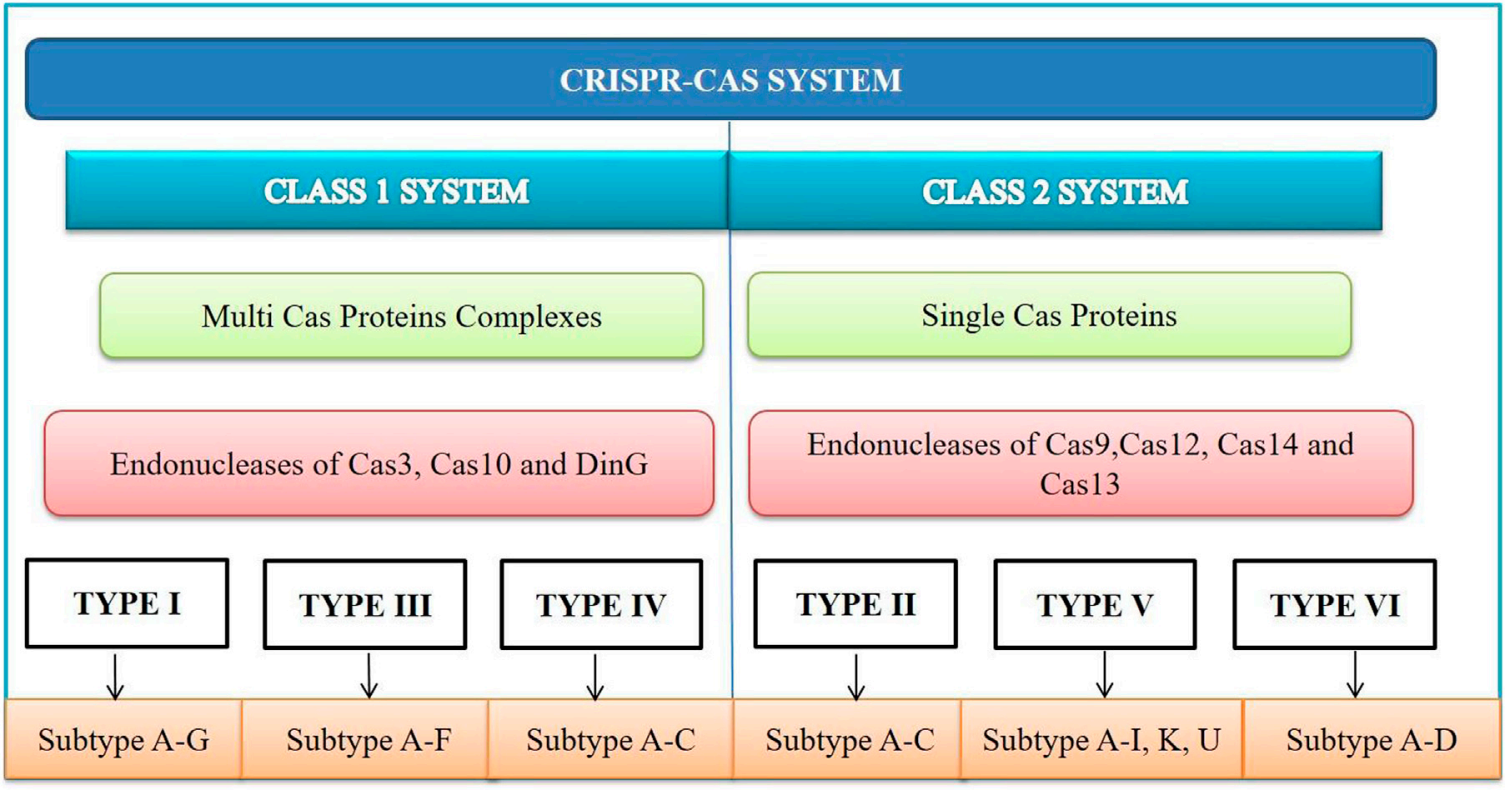
CRISPR-Cas system is divided into Class I and II based on the core Cas proteins. Within each class, there are six types, further subdivided into several subtypes. Class I systems use complexes of multiple Cas proteins, which mediate interference. Class II systems cleave nucleic acids using a single Cas multi-domain effector protein.

According to the article published in *Nature Reviews Microbiology* in 2020, CRISPR-Cas systems are classified into Class I and Class II based on the structural differences in the Cas genes as shown in Figure 2. Class I systems involve multi-protein effector complexes, while Class II systems feature single effector proteins. Six CRISPR-Cas system types and 34 subtypes have been reported (Kato et al., 2022). Among these, the CRISPR-Cas9 type II system is the most popular due to its adaptability and advanced gene editing capabilities. The Cas protein from *Streptococcus pyogenes* (SpCas9) can precisely target specific DNA sequences, making it a valuable tool for gene editing. The CRISPR-Cas system type I, II and III are widely studied types based on the mechanism of target sequence recognition and cleavage (Makarova et al., 2011). Interestingly, the same organism can possess all three of these systems simultaneously.

Class I system contains various loci which encodes Cas1 and Cas2, adaptation module proteins, and different accessory proteins including, CARF (CRISPR-associated Rossmann fold) domain-containing protein, reverse transcriptase, cas4 and many others. Type III and type IV lacks the CRISPR arrays and/or adaptation module genes in their loci. Type I always contains cas3 helicase, and a PAM, varies between subtypes located either 5′ or 3′ of the proto-spacer and needed at both interference and adaptation stage (Makarova et al., 2017).

Type I, type II, and type V mainly targets DNA. In type I system, the signature gene is *Cas3*, that encodes a protein with both nuclease and helicase domains, the effector complex of type I system is cascade complex that targets DNA. The type I CRISPR-Cas system involves the Cas3 enzyme, which is involved in DNA degradation. Cas3 has DNase domain and DNA helicase, allowing it to degrade foreign DNA. This system comprises six subtypes, each characterized by a different number of Cas genes. The CRISPR array is processed by Cas6, forming individual repeat units with the precursor-crRNA. The complex formation between the multi-protein Cas complex and crRNA leads to cascade formation. Base pairing with the complementary sequence of the foreign DNA aids in the defense against viruses (Brouns et al., 2008). Cas3 creates single-stranded breaks in the target DNA and is subsequently degraded by itself (Westra et al., 2012). The type II CRISPR-Cas system is one of the most recognized and studied systems (Gasiunas et al., 2012; Wei et al., 2015). It is distinguished from type I and type III by the activity of its Cas9 nuclease enzyme, which can cause double-stranded, blunt-ended breaks in DNA. The Cas9 enzyme cleaves the target DNA 3 bp upstream of the proto-spacer adjacent motif sequence (PAM). In addition to Cas9, it also contain genes that encode for Cas2 or Cas4 enzymes (Jinek et al., 2012). Cas9 is responsible for processing the crRNA and involves two CRISPR-Cas9 domains with nuclease properties: RuvC and HNH. The HNH domain cleaves the complementary DNA strand of the crRNA, while the RuvC domain cleaves the opposite DNA strand. The type II CRISPR-Cas locus contains a tracrRNA (trans-activating crRNA) located upstream, serving as trans-encoded RNA (Li et al., 2015). In type II, the signature gene Cas9, that encodes a protein that fulfills the roles of the multiple proteins found in the class I effector complexes, Cas9 contains two nuclease domains, RuvC and HNH, and is the only Cas protein required to cleave the target DNA. The discovery of Cas12 also known as Cpf1 gave rise to type V, Cas12 is the only protein which is involved in interference stage and it only contains a RuvC nuclease domain and lacks HNH domain. Type III targets both DNA and RNA, in this type Cas10 gene is the signature gene, a gene encoding a multi-domain protein with nuclease activity involved in the interference stage, Cas10 gene is missing in the subtype III-E the effector complex of type III systems is Cmr complex. Type IV system signature gene is CsfI which is missing in subtype IV-C. Type VI targets RNA, recently discovered Cas13 gene encodes a tracrRNA-independent nuclease which targets RNA (Makarova et al., 2020).

## 5 Recent advances in CRISPR-Cas technology

### 5.1 Exploring CRISPR-Cas13: a breakthrough in RNA editing and its practical applications

Cas13, a class II type VI CRISPR effector, has gained significant attention for its potential applications in RNA editing and various RNA-related technologies. Unlike Cas9, which is commonly used for DNA editing, Cas13 is primarily known for its RNA-targeting capabilities. The Cas13 protein, in association with a CRISPR RNA (crRNA), assembles into an RNA-guided RNA targeting complex. This complex is responsible for recognizing and cleaving single-stranded RNA (ssRNA) targets with high specificity. The binding and cleavage of ssRNA targets are mediated by the interactions between the crRNA and the complementary regions within the target RNA. This unique RNA-guided RNA interference system offers a powerful tool for manipulating and regulating RNA molecules in a precise and programmable manner, with applications ranging from RNA editing in plants and other organisms to diagnostics and beyond. Different Cas13 variants were investigated to determine their catalytic capabilities for RNA virus interference in plants, and it was found that LwaCas13a, PspCas13b, and CasRx variants mediate significant interference activities against RNA viruses in transient assays in *Nicotiana benthamiana*. Furthermore, as compared to the other variants assessed, CasRx induced substantial interference in both transient and stable overexpression assays (Mahas et al., 2019). Aman et al. (2018) assessed the potential of the CRISPR/Cas13a system for RNA interference with RNA viruses in plants, so they designed the CRISPR/Cas13a RNA interference system for in planta applications. In transient experiments and stable overexpression lines of *Nicotiana benthamiana*, CRISPR/Cas13a catalytic activity resulted in TuMV-GFP interference, and Cas13a can convert lengthy pre-crRNA transcripts into functional crRNAs, leading in TuMV interference. Furthermore, CRISPR/Cas13 has since been used to target RNA viruses, for example, potato virus Y (PVY), tobacco mosaic virus (TMV), southern rice black-streaked dwarf virus (SRBSDV), and rice stripe mosaic virus (RSMV) in various plants (Zhan et al., 2019; Zhang et al., 2019). In recent study, CRISPR-Cas13a/d along with CRISPR-Cas12a employed for the diagnostics of infections produced by plant RNA viruses, namely, Tobacco mosaic virus, Tobacco, etch virus, and Potato virus X, in *Nicotiana benthamiana* plants (Marqués et al., 2022). Recently Sharma et al. (2022) successfully showed PDS transcript knockdown in *N. benthamiana*, *A. thaliana*, and *Solanum lycopersicum* making use of LbaCas13a and LbuCas13a by Agrobacterium infiltration. This research also discovered that, in the absence of Cas, the crRNA may induce gene silencing by using the argonaute proteins and the plant RNAi machinery. Type VI CRISPR-Cas systems offer a wide range of applications in diverse organisms through various RNA technologies such asRNA interference, RNA detection, RNA editing, and RNA targeting (Burmistrz et al., 2020; Kordyś et al., 2022; F. Wang et al., 2019). These versatile RNA-based tools have opened up new avenues for research and biotechnological advancements across different fields and organisms. It is important to note that while Cas13 shows great potential for RNA editing and manipulation, the technology is still evolving, and challenges related to specificity, delivery, and off-target effects need to be addressed for plant genome engineering applications (Fu et al., 2014; East-Seletsky et al., 2016). Nevertheless, Cas13 has opened up exciting possibilities for RNA research in plants and other organisms, diagnostics, and therapeutic interventions.

### 5.2 CRISPR interference

**FIGURE 3:**
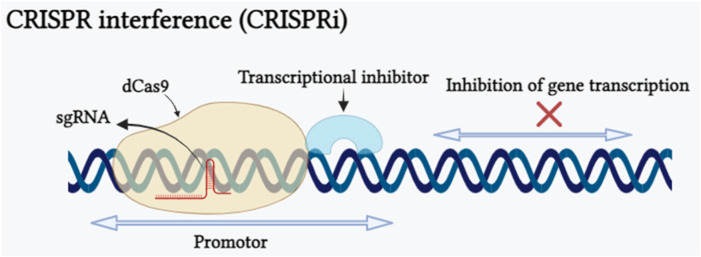
Schematic representation of CRISPRi depicting the CRISPRi-mediated interference of gene expression caused by nuclease-deficient dCas9. The dCas9 transcriptional repression approach is shown. The dCas9-transcriptional inhibitor is directed to selected genetic loci (promoter sequence of the targeted gene) by a sgRNA with a spacer complementary to the targeted genomic locus near to a PAM motif. Binding of the dCas9:sgRNA complex upstream of the transcription start site restricts recruitment of the RNA polymerase, whilst assembly at a downstream position inhibits transcription elongation (Created with BioRender.com).

After 1 year of using CRISPR-Cas as a gene editing tool, scientists have uncovered a novel RNA-guided DNA binding system called CRISPR interference (CRISPRi), derived from *Streptococcus pyogenes*, which can be adapted as an innovative method for suppressing the transcription of any gene (Qi et al., 2013). An advantageous feature of CRISPRi is its ability to simultaneously suppress multiple target genes and its effects are reversible. This is accomplished by targeting precise genomic sequences by modulation of the transcription process without altering the underlying DNA sequence. CRISPRi is formed by combining a catalytically inactive Cas9 protein called dCas9 with a guide RNA designed to bind to the promoter region of the target gene, allowing it to interrupt transcriptional elongation, RNA polymerase binding, or transcription factor binding processes (Xu et al., 2023). CRISPRi-mediated transcriptional repression of the gene has been shown in (Figure 3). CRISPRi offers distinct advantages over other gene editing tools such as ZFN and TALEN. The repetitive nature of custom zinc-finger or TALE proteins makes the construct development an expensive and time-consuming process, limiting the creation of protein libraries (Klug, 2010). In contrast, CRISPRi relies on single-guide RNA with a gene-specific 20-nucleotide-long complementary region, making it a cost-effective and simple method for oligo-based gene regulation. In plant research, the SRDX domain, also known as SUPERMAN REPRESSION DOMAIN X, has been widely utilized in CRISPRi. Notably, SRDX is the only reported transcriptional repressor domain (TRD) that has proven effective when combined with dCas9 protein in plant systems. This fusion enables precise and targeted gene repression, making SRDX a valuable tool to manipulate gene expression for multiple applications such as crop improvement and functional genomics. However, the incomplete repression of SRDX repression domain limits the application of CRISPRi system. Recently Xu et al. (2023) proved that three functional TRDs, namely, DLN144 (a DLN hexapeptide motif), DLS and MIX (obtained by the conjugation of DLN144 and SRDX), offer additional possibilities for using CRISPRi in plants. This research also provides insight into the development of more robust TRDs by merging several effective repressor domains individually which will enable the application of CRISPRi when there is a demand for higher repression efficiency. Tang et al. (2017) targeted miR159b for transcriptional repression using CRISPRi and showed tenfold reduction in miR159b transcription in Arabidopsis, similarly, in the rice plant they demonstrated repression of OsPDS, OsDEP1 and OsROC5 genes. In some studies carried out in Arabidopsis, researchers have used modified forms of CRISPR/dCas9 components such dCas9-3xSRDX and dCas9-TALE-SRDX targeting CFTS64 and RD29-LUC genes respectively for transcriptional repression (Mahfouz et al., 2012; Lowder et al., 2015). Furthermore, two modified forms of CRIPSRi dCas9-SRDX and dCas9-BRD demonstrated transcriptional repression of reporter gene pNOS::LUC in *Nicotiana benthamiana* (Piatek et al., 2015; Vazquez-Vilar et al., 2016). Hence, CRISPRi can be concluded as a powerful gene editing tool that allows precise and reversible gene repression, but it has its own limitations and challenges such as CRISPRi can influence genes that are nearby to the target gene and PAM sequence requirement restrict the potential target sequences.

### 5.3 CRISPR activation

**FIGURE 4:**
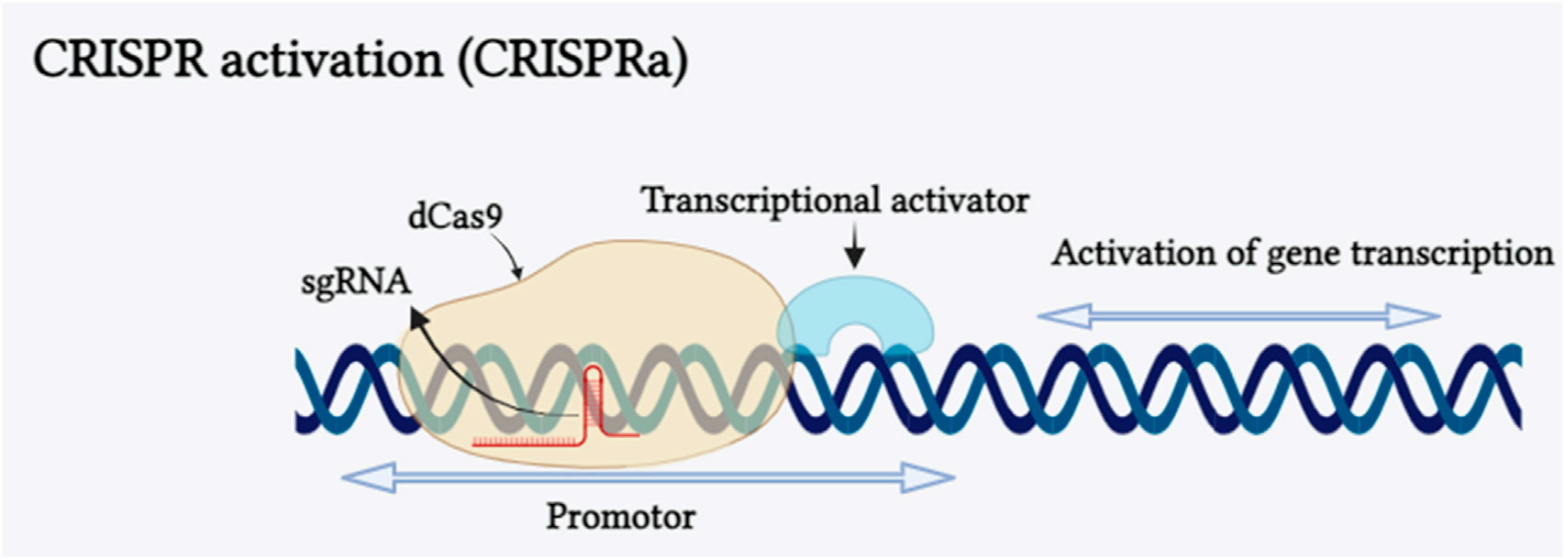
CRISPR activation inducing gene expression is illustrated schematically. CRISPR activation (CRISPRa) is a particular type of CRISPR tool that employs customized forms of CRISPR effectors lacking endonuclease activity, as well as adding transcriptional activators on dCas9 or guide RNAs (gRNAs). The binding of the dCas9/sgRNA complex to a promoter sequence of targeted gene results in transcriptional activation of the gene. (Created with Biorender.com).

CRISPR activation, often referred to as CRISPRa, represents a modified form of CRISPR technology. Bikard et al. (2013) fused catalytically dead dCas9 protein with omega (ω) subgroups (rpoZ) in *Escherichia coli*, resulting in the formation of dCas9-ω complex. This complex increased the transcriptional level of the reporter gene up to 2.8 fold. Based on this principle a RNA-guided activation, “CRISPRa” was composed by the fusion of dCas9 with a transcriptional effector to modulate the expression of target gene. The mechanism of CRISPR activation inducing gene expression has been shown in (Figure 4). CRISPR activation strategies have been extensively employed in animal cells, their application in plants is still limited. In plants, this approach is predominantly employed in model plant species like *Arabidopsis thaliana* and *Oryza sativa*. The initial transcription activation experiment was conducted on model plant species *Nicotiana benthamiana* and *Arabidopsis thaliana* to assess the capability of dCas9-VP64. As a result, dCas9-VP64 showed limited activation activity when a single guide RNA was designed in the vector. Subsequently, when multiple sgRNAs were utilized to target the same gene promoter, it led to a greater gene activation activity (Piatek et al., 2015; Vazquez-Vilar et al., 2016). Endogenous genes transcription can be increased by activating CRISPRa and multiple gene activation is also possible with this by fusing with different transcription activation domains (TADs). dCas9-SunTag System, dCas9-VPR System, dCas9-TV System are resulted from the fusion of transcriptional activation effectors with dCas9 and scaffold RNA (scRNA) system, SAM System, CRISPR-Act 2.0 System, CRISPR-Act3.0 System are the common method for modification of gRNA into a scaffold and recruitment of transcriptional activators. CRSIPRa for gene activation have been employed in many plants, including in *Oryza sativa* CRISPRa targeted Os03g01240 2.0, OsER1 and Os04g39780 genes and found 2.0, 62.0, 4.0 fold increase in the transcription (Li et al., 2017; Lowder et al., 2018), in Arabidopsis pWRKY::luciferase, AtWRKY and AtCLAVATA3 genes showed 6.7, 11.7 and 100 fold increased transcript (Li et al., 2017; Papikian et al., 2019), and in *N. benthamiana* pNOS::luciferase and NbPDS genes transcription was increased 3.0 and 3.4 fold (Piatek et al., 2015; Selma et al., 2019). CRISPRa has made a huge impact on biotechnology in medical science, agriculture, epigenetic regulation research and also in plant defense mechanism against pathogen but this method is only used in model plants (Didovyk et al., 2016; Paul and Qi, 2016; Lowder et al., 2018; Ding et al., 2022). The main challenge of CRISPRa is long sequences of multiple gRNAs increase in number of gRNA sequences leads to limited dCas9 competition between various gRNAs causes unpredictability in the regulation of target genes by gRNAs (Mao et al., 2018) and variations in the efficiency of target gene editing (Zhang and Voigt, 2018).

### 5.4 Base editors and prime editors

Most genetic variations that cause diseases and are agriculturally significant are single-base polymorphisms that require precise gene editing technologies for efficient repair. However, homology-directed DSBs are not effective in differentiated plant cells. Two newly developed genetic engineering techniques have emerged to address this issue: base editing and prime editing, which can induce precise modifications in target regions. These techniques have been applied successfully in a wide range of plant species. Base editing allows effective and precise point mutations at specific target sites, necessitating donor DNA templates or DSBs (Rees and Liu, 2018). Currently, three categories of base editors are commonly used: cytosine base editor (CBE) for C:G to T:A transitions, C-to-G base editors (CGBEs) for inducing G:C to C:G transitions, and adenine base editors (ABE) for A: T to G:C transitions (Li et al., 2022). These three editor types have been used extensively for gene repair and functional annotation due to their effectiveness and simplicity in precise base editing. Recent success in the improvement of crops is summarized in Table 2.

**TABLE 2:** Overview of recent successes in crop improvement using base editors and prime editors.

Editor type	Target gene	Cultivar	Base editing system	Editing efficiency	Transformation method	References
Base editors						
BE2	*NRT1.1B*, *SLR1*	*Oryza sativa*	APOBEC1-XTEN-Cas9(D10A)	≤13.3%	*Agrobacterium-*mediated transformation	Lu and Zhu (2017)
BE3	*OsDEP1*	*Oryza sativa*	xCas9(D10A)-rAPOBEC1	≤30%	*Agrobacterium-*mediated transformation	Zhong et al. (2019)
BE3	*OsCERK1*, *OsSERK1*, *OsSERK2*, *ipa1*	*Oryza sativa*	APOBEC1-XTEN-Cas9n-UGI-NLS	10%–38.9%	*Agrobacterium-*mediated transformation	Ren et al. (2017)
BE3	*OsNRT1.1B*, *OsSPL14*, *OsCDC48*	*Oryza sativa*	pnCas9-PBE	43.48%	*Agrobacterium-*mediated transformation	Zong et al. (2017)
ABE	*OsCDC48*, *OsALS, OsDEP1*, *OsAAT*, *OsNRT1.1B*, *OsEV*, *OsOD*, *OsACC*	*Oryza sativa*	PABE	3.2%–59.1%	*Agrobacterium-*mediated transformation	Li et al. (2018)
APOBEC1-CBE2/CBE3	*GhCLA*, *GhPEBP*	*Gossypium herbaceum*	APOBEC1-XTEN-nCas9-UGI	0%–57.78%	*Agrobacterium-*mediated transformation	Qin et al. (2020)
hAID-CBE3	*OsAOS1*, *OsFLS2*, *OsJAR2*, *OsCOI2*, *OsPi-D2*, *OsJAR1*	*Oryza sativa*	hAID-XTEN-nCas9	8.30%–73.30%	*Agrobacterium-*mediated transformation	Ren et al. (2018)
hAID-CBE3	*OsCOI2*, *OsBSR*, *OsMPK13*, *OsGS1*, *OsGSK4*	*Oryza sativa*	hAID-XTEN- nSpRY-UGI	26.00%–34.15%	*Agrobacterium-*mediated transformation	Xu et al. (2021)
hAID-CBE3	*OsMPK16*, *OsCPK6*, *OsCPK5*, *OsMPK9*, *OsMPK17*, *OsCPK7*, *OsMPK15*, *OsCPK8*	*Oryza sativa*	hAID-nScCas9-UGI	2.56%–97.92%	*Agrobacterium-*mediated transformation	Wang et al. (2020)
				0%–95.83%		
PmCDA1-CBE2/CBE3/CBE4	*OsDEP1*, *OsCDC48*, *OsGS3*	*Oryza sativa*	PmCDA1-xCas9-UGI	0%–21.10%	*Agrobacterium-*mediated transformation	Zhong et al. (2019)
PmCDA1-CBE2/CBE3/CBE4	*OsPDS*	*Oryza sativa*	PmCDA1-nSpCas9-NG-UGI	3.50%–56.30%	*Agrobacterium-*mediated transformation	Zhong et al. (2019)
PmCDA1-CBE2/CBE3/CBE4	*OsWaxy*, *OsEUI1*	*Oryza sativa*	PmCDA1-nScCas9+ +-UGI-UGI	8.3%–86.1%	*Agrobacterium-*mediated transformation	Liu et al. (2021)
PmCDA1-CBE2/CBE3/CBE4	*SlALS*	*Solanum lycopersicum*	nCas9-NG-PmCDA1-UGI	32.00%	*Agrobacterium-*mediated transformation	Veillet et al. (2020)
		*Solanum tuberosum*				
ABE7.10	*OsACC*, *OsALS, OsOD*, *kjjjOsCDC48*, *OsAAT*, *OsDEP1*, *OsNRT1.1B*, *OsEV*, *TaEPSPS*, *TaGW2*, *TaDEP1*	*Oryza sativa*	TadA-TadA7.10-nCas9(D10A)	3.20%–59.10%	*Agrobacterium-*mediated transformation/particle bombardment	Li et al. (2018)
		*Triticum*				
ABE7.10	*Tms9-1*, *OsCPK9*, *OsMPK15*, *OsMPK14*, *OsCPK10*	*Oryza sativa*	TadA-TadA7.10-nScCas9	50.00%–94.12%	*Agrobacterium-*mediated transformation	Wang et al. (2020)
ABE-P1S	*SLR1*, *OsDEP1*, *OsACC1*, *OsNRT1.1B*, *OsSPL14*, *OsSERK2*	*Oryza sativa*	TadA7.10-nSaCas9, TadA7.10-nSpCas9	4.50%–96.30%	*Agrobacterium-*mediated transformation	Hua, Jiang, et al. (2020)
				0%–61.10%		
ABE8e	*OsEPSPS*, *OsALS*, *OsWaxy*	*Oryza sativa*	TadA8e (V106W) nCas9	4.00%–100.00%	*Agrobacterium-*mediated transformation	Wei et al. (2021)
			TadA8e (V106W)- nCas9-NG	0%–100.00%		
pDuBE1	*OsALS*, *OsBADH2*, *OsLAZY1*, *OsPDS*	*Oryza sativa*	TadA8e-nCas9-CDA1-UGI	0.40%–87.60%	*Agrobacterium-*mediated transformation	Xu et al. (2021)
ABE8e	*OsLF1*, *OsSPL14*, *OsSPL7*, *OsCKS2*, *OsIAA13*, *OsGBSSI*, *OsEUI1*, *OsTS*	*Oryza sativa*	TadA8e-DBD-nSpRY, TadA8e-DBD-nSpG, and TadA8e-DBD- nCas9-NG	0%–90.50%	*Agrobacterium-*mediated transformation	Tan et al. (2022)
				0%–92.50%		
				0%–100.00%		
ABE9	*OsJAR1*, *OsWRKY45*, *OsMPK6*, *OsGS1*, *OsSERK2*, *OsDEP2*, *OsETR2*, *OsGSK4*, *OsALS1*, *OsMPK13*	*Oryza sativa*	TadA9-XTEN- nScCas9, TadA9-XTEN-nSpRY, TadA9-XTEN- nSpCas9-NG, andTadA9-XTEN- nSpCas9	0%–97.92%	Agrobacterium-mediated transformation	Yan et al. (2021)
				0%–100.00%		
				0%–37.50%		
				0%–68.75%		
DdCBE	*16s rRNA*, *rpoC1*, *psbA*	*Arabidopsis thaliana*	cTP-TALE-L-nDdA- UGI-cTP-TALE-R-cDdA-UGI, PTP-TALE-L-nDdA-UGI-PTP-TALE-R-cDdA-UGI	0%–86.40%	*Agrobacterium-*mediated transformation	Nakazato et al. (2021)
DdCBE	*OspsaA*	*Oryza sativa*		97.50%	*Agrobacterium-*mediated transformation	Li et al. (2021)
ABE7.10-nCas9	*35S, AtUbi, AtRPS5A, SlRPS5A1 (Solyc11g042610)*, *SlRPS5A2 (Solyc10g078620)*, *SlTCTP (Solyc01g099770) and SlEF1α (Solyc06g005060)*	*Solanum lycopersicum* *Glycine max*	*pSlEF1α*-ABE-nCas9, *pSlEF1α*-ABE-XNG-nCas*9* or *pSlEF1α*-ABE-nCas9-NG	15%–40%	*_*	Niu et al. (2023)
Prime editors						
PE2						
PE2	*OsALS*, *OsACC*	*Oryza sativa*	1–3 bp substitution	Desired %	*Agrobacterium-*mediated transformation	Jiang et al. (2022)
				1.00%–7.60%		
				Undesired %		
				0.00%		
PE2	*OsALS*, *OsIPA1*, *OsTB1*	*Oryza sativa*	2–4 nucleotide substitution, 2 bp insertion	Desired %	*Agrobacterium-*mediated transformation	Butt et al. (2020)
				0.00%–2.04%		
pPE2	*HPTII*, *OsPDS, OsACC*, *OsWx*	*Oryza sativa*	1 bp substitution,1–3 bp insertion	Desired %	*Agrobacterium-*mediated transformation	Xu et al. (2020)
				0.00%–59.90%		
pPE2	*OsCDC48*, *OsPDS*, *OsALS*, *OsACC*	*Oryza sativa*	1–2 bp substitution, 1 bp insertion	Desired %	*Agrobacterium-*mediated transformation	Li et al. (2022)
				0.00%–29.17%		
pPE2-engineered pegRNA with mpknot)	*OsPDS*, *OsALS*, *OsCDC48*	*Oryza sativa*	1–2 bp substitution, 1 bp insertion	Desired %	*Agrobacterium-*mediated transformation	Li et al. (2022)
				10.42%–25.00%		
Sp-PE2	*GFP*	*Oryza sativa*	2 bp substitution	Desired %	*Agrobacterium-*mediated transformation	Hua et al. (2020)
				15.60%		
PPE	*OsALS*	*Oryza sativa*	2 bp substitution	Desired %	*Agrobacterium-*mediated transformation	Zong et al. (2022)
				2.10%		
pZ1WS (driven by the composite promoter CaMV35S-CmYLCV-U6)	*ZmALS2*, *and ZmALS1*	*Zea mays*	2–3 bp substitution	Desired %	*Agrobacterium-*mediated transformation	Jiang et al. (2020)
				4.80%–53.20%		
pPE2 (an engineered pegRNA with evopreQ1)	*OsALS*, *OsACC, OsCDC48, OsPDS*	*Oryza sativa*	1–2 bp substitution, 1 bp insertion	Desired %	*Agrobacterium-*mediated transformation	Li et al. (2022)
				2.08%–50.00%		
ePPE (replaced M-MLV-RT to M-MLV-RT-ΔRNaseH)	*OsALS*	*Oryza sativa*	2 bp substitution	Desired %	*Agrobacterium-*mediated transformation	Zong et al. (2022)
				11.30%		
PE3						
PE3	*HPTII*, *OsEPSPS*	*Oryza sativa*	3–7 bp substitution	Desired %	Particle bombardment	Li et al. (2020)
				2.22%–9.38%		
Sp-PE3	*APO1*, *GFP*, *OsALS*	*Oryza sativa*	1–2 bp substitution	Desired %	*Agrobacterium-*mediated transformation	Hua et al. (2020)
				0.00%–17.10%		
PE-P2 (nCas9-M-MLV-T2A-hpt)	*OsACC*, *OsDEP1, OsALS*	*Oryza sativa*	1–4 bp substitution	Desired %	*Agrobacterium-*mediated transformation	Xu et al. (2020)
				1.70%–26.00%		
				Undesired %		
				0.00%–8.00%		
PE-P2-RT-S (N- terminal M-MLV + one target mutation in RTT)	*OsPSR1OsDEP1*, *OsSD1*, *OsALS*, *OsACC*, *OsGRF4*, *OsChalk5*, *OsWaxy*, *OsEPSPS*, *OsCold1*, *OsGS3*, *OsChalk5*	*Oryza sativa*	1 bp substitution	Desired %	*Agrobacterium-*mediated transformation	Xu et al. (2022)
				0.00%–61.40%		
				Undesired %		
				0.00%–15.00%		
pCXPE03 (the RPS5A promoter derives that)	*SlGAI*, *SlALS*, *SlPDS*	*Solanum lycopersicum*	2 bp substitution, 2 bp insertion	Desired %	*Agrobacterium-*mediated transformation	Lu et al. (2021)
				0.00%–6.70%		
PE3	*OsSPL14*, *OsDHDPS*, *OsNR2*	*Oryza sativa*	2–3 nucleotide substitution	Desired %	Particle bombardment	Li et al. (2022)
				0.00%–1.00%		
PE3	*OsALS*, *OsACC*, *OsEPSPS*	*Oryza sativa*	1–3 bp substitution	Desired %	*Agrobacterium-*mediated transformation	Jiang et al. (2022)
				1.30%–70.30%		
				Undesired %		
				9.00%–37.90%		
PPE3	*OsCDC48*, *OsALS*	*Oryza sativa* and *Triticum*	1–3 bp substitution, 6 bp deletion	Desired %	*Agrobacterium-*mediated transformation	Lin et al. (2020)
				2.60%–21.8%		
pPE3	*OsWx*, *OsACC*	*Oryza sativa*	1 bp substitution	Desired %	*Agrobacterium-*mediated transformation	Xu et al. (2020)
				0.00%–16.70%		
PE-P1	*OsALS*, *OsDEP1*, *OsACC*	*Oryza sativa*	1–4 bp substitution	Desired %	*Agrobacterium-*mediated transformation	Xu et al. (2020)
				0.00%–1.40%		
				Undesired %		
				0.00%		
pPE3b	*OsACC*	*Oryza sativa*	1 bp substitution	Desired %	*Agrobacterium-*mediated transformation	Xu et al. (2020)
				6.25%		
PE4						
PE4	*OsACC*, *OsALS*	*Oryza sativa*	1–3 bp substitution	Desired %	*Agrobacterium-*mediated transformation	Jiang et al. (2022)
				5.20%–27.10%		
				Undesired %		
				0.00%–2.10%		
PE5						
PE5	*OsACC*, *OsALS*, *OsEPSPS*	*Oryza sativa*	1–3 bp substitution	Desired %	*Agrobacterium-*mediated transformation	Jiang et al. (2022)
				1.60%–64.10%		
				Undesired %		
				6.40%–18.30%		
PPE2	*OsDLT*	*Oryza sativa*	1–2 bp substitution	Desired %	*Agrobacterium-*mediated transformation	Xue et al. (2023)
				2.5%–84.9%		

#### 5.4.1 Cytosine base editor (CBE)

**FIGURE 5:**
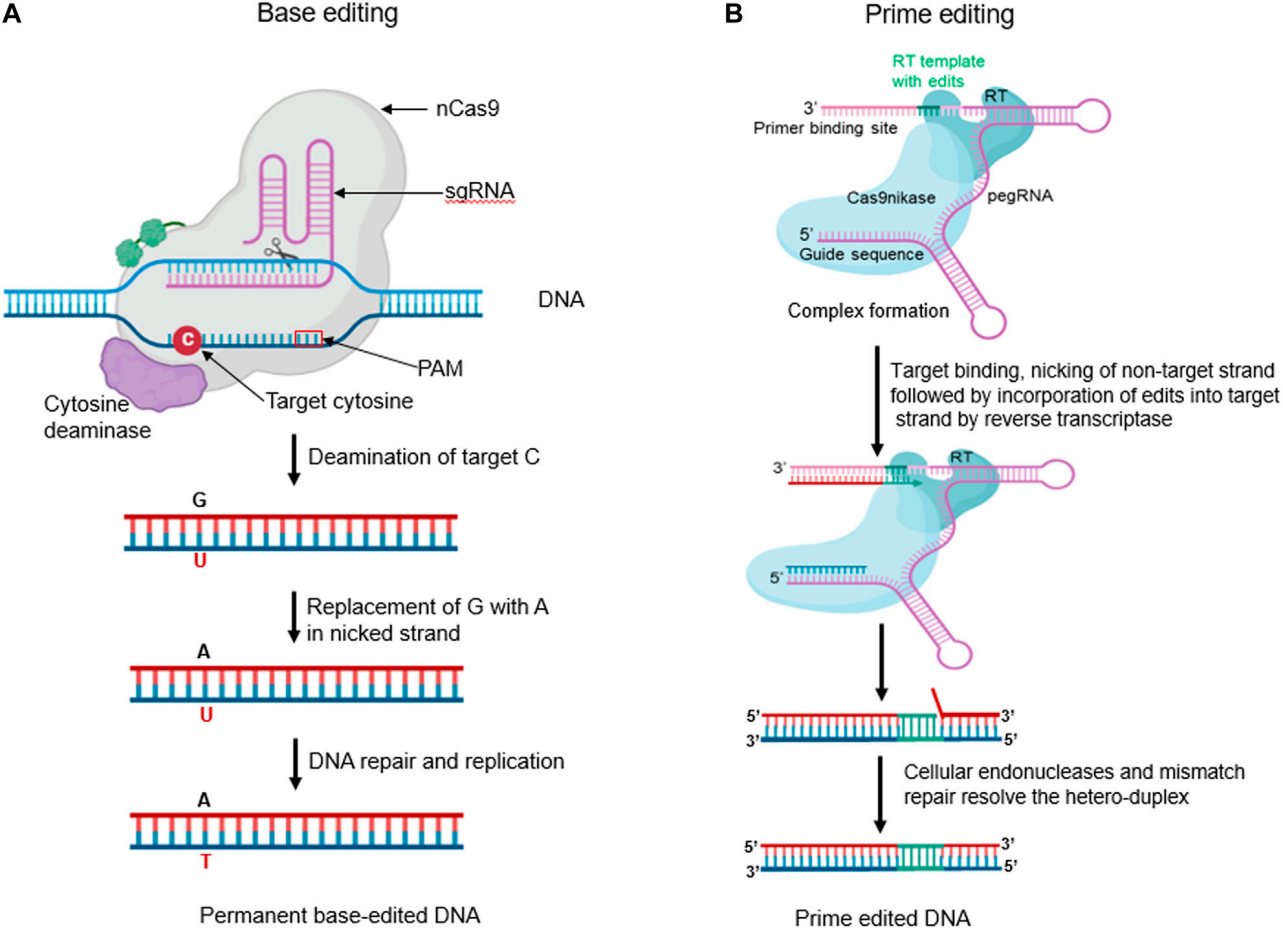
**(A)** Base editors (BEs) are innovative fusion proteins engineered by combining a nickase Cas9 (nCas9) with deaminase domain(s). Cytosine base editor (CBE) can convert C to U in a single strand. The resulting U:G hetero duplex can then be converted to a T:A base pair following DNA replication or repair (Created with BioRender.com). **(B)** The Prime Editor (PE) is a remarkable engineered fusion protein, meticulously crafted by combining the properties of a nickase Cas9 (nCas9) and a reverse transcriptase (RT). The nCas9 creates an SSB on the non-target strand. The released 3′ end then hybridizes to the 3′ end of the prime-editing guide RNA (pegRNA) and is reverse transcribed by the RT domain. The reverse transcription incorporates the edits encoded in the pegRNA into the newly synthesized DNA strand. Equilibration between the edited 3′ flap and the unedited 5′ flap, endogenous 5′ flap cleavage and ligation, and DNA repair results in the stable incorporation of the desired edit in the genome. (Created with BioRender.com).

Cytosine base editors (CBEs) are gene editing tools that allow for precise C:G to T:A conversions at specific target sites. This approach is composed of fusion proteins engineered by combining nickase Cas9 (nCas9) with cytosine deaminase domain. A nuclease-deficient CRISPR system guides CBEs. Deamination of cytosine at the target site results in uracil, which ultimately changes a C:G base pair (bp) to T:A bp without generating a DNA double strand break (Figure 5A). Over the years, different generations of CBEs have been developed to improve their efficiency and specificity. Komor et al. (2016) developed the first-generation cytosine base editor (CBE1) by merging cytidine deaminase (rAPOBEC1) to the N-terminus region of a defective dCas9. The fusion results in cytosine deamination to uracil on the non-target DNA strand, which is then recognized as T by the cell replication machinery, resulting in a C:G to T:A transition. However, CBE1’s efficiency is limited by the action of the base excision repair enzyme, uracil DNA N-glycosylase (UNG), which can reverse the conversion of C-G to T-A and remove U from the C-G to U-A pair. As a result, Lee et al. (2023) developed the second-generation base editor (CBE2), which includes a uracil DNA glycosylase inhibitor structurally bound to the C-terminus of CBE1. CBE2 increased the editing efficiency three-fold, resulting in fewer unacceptable indels (<0.1%). Doman et al. (2020) developed the third-generation base editor (CBE3) by fusing the nCas9 (D10A) nickase to the uracil DNA glycosylase inhibitor and rAPOBEC1. While CBE3 cannot cleave dsDNA, it induces cellular repair by creating a nick in the target strand. Yu et al. (2020) developed the fourth-generation base editor (CBE4) to improve deamination activity by fusing two uracil DNA glycosylase inhibitors at the C-terminus of cas9 nickase. CBE4 exhibits improved base editing efficiency, decreasing the C:G or A transversion frequency by 2.3 times compared to CBE3.

CBEs have been applied successfully in various plant species for different applications. For example, marker-free tomato plants were produced with homozygous heritable DNA substitutions. CRISPR-Cas9 cytidine deaminase targeted base modification fusion in rice facilitated multiple herbicide-resistant point mutations through multiplexed editing with herbicide selection (Shimatani et al., 2017). Zong et al. (2017) used a nr67Cas9-cytidine deaminase fusion in regenerated maize, rice, and wheat plants and protoplasts to successfully convert cytosine to thymine from positions 3 to 9 within the proto-spacer at a modification frequency of up to 43.48%. In rice, wheat, and potato, A3A-PBE (human APOBEC3A) successfully induced efficient C to T conversions at multiple endogenous genomic loci with various sequences within a 17 bp deamination window (Zong et al., 2018). Watermelon-generated plants with mutations in C to T conferred base editing with 23% efficiency in T0 plants, and CRISPR-Cas9-mediated base editing was used to develop a non-GM herbicide-resistant watermelon cultivar (Tian et al., 2018). Researchers have developed a base editor system (GhBE3) comprising a cytidine deaminase domain linked to UGI and nCas9 to target two essential genes, GhPEBP and GhCLA, in allotetraploid cotton (Qin et al., 2020). The system generated a C:G to T:A conversion at three target sites with a modification efficiency of 26.67%–57.78% with no off-target impacts (Qin et al., 2020). Jin et al. (2020) created two effective (CBE) variants in rice (A3BctdVHMBE3 and A3BctdKKRBE3) that eliminated sgRNA-independent DNA off-target edits and generated fewer multiple C edits at their target sites.

In addition, CBEs are a promising tool for modifying cis-regulatory regions to modify gene expression; using this method, researchers successfully increased the sugar content in strawberries (Xing et al., 2020). An engineered form of hAID cytosine deaminase was used to develop a toolkit of base editors that can successfully downregulate target gene activity in plants by generating an upstream open reading frame and evaluating the loss-of-function of non-coding genes such as microRNA sponges (Xiong et al., 2022).

#### 5.4.2 Adenine base editors (ABE)

Adenine base editors (ABEs) are genome editing tools that facilitate precise A:T to G:C conversions at specific target sites. Unlike CBEs, which use cytidine deaminases, ABEs employ adenosine deaminases for their editing mechanisms. ABEs have three primary components: Cas9 nickase, sgRNA, and mutant transfer RNA adenosine deaminase (TadA) (Rees et al., 2019). The deamination of adenine in ABEs leads to the conversion of adenine to inosine, which is recognized as G and linked to C in the replication process. The first-generation adenine base editor (ABE 1.2) was developed by fusing two important components to the nCs9, such as the nuclear localization signal (NLS) with the C-terminal region of nCas9 and the N-terminal region of nCas9 with the linker XTEN with the heterodimer TadA-TadA (Kim et al., 2019). Over time, ABEs have undergone numerous optimizations and modifications to increase their editing efficiency, including various TadA mutations that connect the nCas9 (D10A) C-terminal region to the TadA (2.1) domain, change the linker length between nCas9 (D10A) and TadA (2.1), and use a TadA inactivated N-terminal subunit. Through directed evolution and protein engineering, the seventh generation of ABEs, was generated (Koblan et al., 2018; Gaudelli et al., 2020). ABE7.10 exhibited higher product purity (often 99.9%) and very few indels (often ≤0.1%) (Gaudelli et al., 2017). Later, ABEmax incorporated an extra NLS at both ends of ABE7.10, with the editing efficiency at most target sites <50% (Hua, Tao, et al., 2020; Yan et al., 2021). In rice, ABEmax introduced an A:T to G:C conversion in OsACC (Yan et al., 2021), OsMPK6, OsSERK2, and OsWRKY45 at frequencies of 17.6%, 32.1%, and 62.3%, respectively. Compared to the commonly used TadA TadA7.10nCas9 (D10A) fusion, a more straightforward base editor (ABE-P1S) that contains TadA7.10nCas9 (D10A) revealed much greater editing efficiency in rice (Hua, Tao, et al., 2020). Recently, TadA8e, a more effective adenine deaminase variation artificially evolved from TadA7.10, was used to develop ABE8e (Gaudelli et al., 2017; Richter et al., 2020). Compared to ABE7.10, ABE8e deaminates the target base almost 1,000 times faster, significantly improving the correction efficiency of A to G. The V106W mutation was inserted in TadA8e to decrease off-target effects (Richter et al., 2020). Furthermore, rABE8e (rice ABE8e), a high-efficiency ABE, was constructed in rice by coupling the codon-optimized monomeric TadA8e and bis-bpNLS (NLS at the N and C terminus) (Wei et al., 2021). Compared to ABEmax in rice, the rABE8e significantly increased the editing efficiency on the two target sequences: NGG-PAM and NG-PAM. A higher homozygous ratio substitution was obtained during the editing window, precisely at locations A5 and A6, and rABE8e had approximately 100% editing efficiency for most targets (Wei et al., 2021). A newly efficient ABE toolbox (PhieABE) was constructed based on hyTadA8e by fusing a single-stranded (DBD) DNA-binding domain and TadA8e. Compared to ABE8e systems, PhieABE shows substantially higher broader editing windows and base editing activity (Tan et al., 2022). Adenosine deaminase TadA9, a more effective variant, was finally obtained in rice by incorporating two mutations, V82S and Q154R, into TadA8e (Yan et al., 2021). Later, a CRISPR-based technology, SWISS (simultaneous and wide editing induced by a single system), was designed to facilitate simultaneous and multiplexed base editing in rice (Li et al., 2020). The SWISS technique uses a sgRNA scaffold with an attached RNA aptamer to recruit binding proteins bonded with adenine deaminase and cytidine deaminase to nCas9 (D10A). This method simplifies the production of A:T to G:C and C:G to T:A alterations at specific gene target sites within the editing window. However, the efficiency of SWISS in plants requires further improvement (Li et al., 2020).

#### 5.4.3 C to G base editors (CGBEs)

While ABEs and CBEs are widely used for base transitions in various organisms, they do not induce base transversions. DNA glycosylase initiates BER by removing U from DNA double strands in many organisms, including plants and bacteria. Recently, a C-to-G base editor called CGBE was discovered in bacteria and mammalian cells to facilitate C-to-G editing in these organisms. CGBEs contain a nCas9 (D10A) and uracil DNA glycosylase (UNG) a variant of rAPOBEC1’s cytidine deaminase (R33A) (Kurt et al., 2021; Zhao et al., 2021). In rice, the OsCGBE03 base editor was developed by optimizing the UNG codon to achieve efficient C-G editing. The efficiency of OsCGBE03 was tested on five endogenous genes (OsALS1, OsIPA1, OsSLR1, OsbZIP5, and OsNRT1.1B), resulting in a 21.3% average frequency of C-to-G base transversions in rice (Tian et al., 2022).

Another variant of CGBE [TadA8e-derived C-to-G BE (Td-CGBE)] was created by introducing an N46L mutation in the TadA-8e that eliminated adenine deaminase activity. Td-CGBE can effectively and precisely edit C-G to G-C conversions (Chen et al., 2022). However, further research is needed to assess its viability in plants. CGBE offers a powerful tool for creating maximum base substitution types in precise crop breeding, developing new germplasm resources, and expanding the base editing toolbox for genetic research and agricultural applications.

Due to the limitations and its poor efficiency, it became necessary to create more advanced versions of base editors. To optimize and improve the range and effectiveness of editing, further development of the base editors is advised. This entails improving the technology to combat by stander mutation production and off-target consequences. In addition, effective sgRNA creation using computational tool is another strategy for getting around some of these restrictions. Despite this, base editing can be utilized to precisely alter crops for sustainable production in the face of recent worldwide shifts.

#### 5.4.4 Repair for base editing in RNA

Base editing in RNA can be repaired through various approaches, depending on the nature of the undesired edit or mutation. RNA editing mediated by cytosine and adenosine deaminases leads to alterations in the identity of the edited bases. Cytosine bases are transformed into uracil base, whereas adenines are converted into inosines. When these edits occur within the coding regions of mRNA, they can potentially modify the RNA’s coding capacity, depending on which codon is affected (please refer to Figure 5A). The recoding potential of nucleotide deaminases has garnered recent interest due to their capability to rectify genetic mutations, either by reversing the mutation itself or by influencing processes like RNA splicing. Zhang’s research group developed two CRISPR-based systems that utilize RNA-targeting Cas13 proteins in combination with ADAR2 to achieve precise base editing of specific RNAs. The first system, known as REPAIR, utilizes the active domain of ADAR2 to achieve programmable adenosine-to-inosine (A-to-I) editing. The second system, RESCUE, employs a modified ADAR2 enzyme to enable additional cytidine-to-uridine (C-to-U) editing (Cox et al., 2017; Abudayyeh et al., 2019). It is important to note that repairing RNA edits is an evolving field, and the choice of strategy may depend on the specific nature of the edit, the cell type, and the desired outcome. Additionally, the safety and ethical considerations of these approaches must be carefully evaluated, especially when applied to therapeutic RNA editing.

### 5.5 Prime editors

Prime editing is a genome editing technique that allows for the precise generation of small indels, all types of single or multiple base substitutions (transversions or transitions), and their combinations at a target site in mammalian cells without the need for a donor DNA template or DSBs (Anzalone et al., 2019). The method uses a catalytically impaired nCas9 (H840A) fused at the C-terminal region with an M-MLV-RT. The prime editing guide RNA (pegRNA) comprises three components: a sgRNA targeting a specific site, an RT encoding the desired edit as a template, and a primer binding site (PBS) that initiates reverse transcription. The protein complex binds to the target DNA and creates a nick in the non-target strand. The 3′ DNA terminal then hybridizes with the PBS and initiates reverse transcription as shown in (Figure 5B). After DNA replication and repair, the desired mutation is copied into the genomic DNA (Li et al., 2022).

Multiple generations of prime editors have been created, each with improved modification efficiency and product purity through optimized guide RNA designs and protein engineering. For example, PE2 incorporates a mutated M-MLV-RT with six mutations to enhance prime editing efficiency (Li et al., 2022). PE3 uses an additional nicking sgRNA to direct cleavage on the unedited strand at varying distances from the pegRNA nicks, maximizing modification efficiency. PE3b uses a specific sgRNA that complements the edited DNA strand and induces another nick after the altered flap is integrated into the genomic DNA, reducing unwanted indels (Nelson et al., 2022; Sretenovic and Qi, 2022). PE5 and PE4 were developed by fusing a dominant negative mismatch repair (MMR)protein cdx (MLH1dn) to the C-terminal part of PE3 or PE2, avoiding DNA mismatch repair. PEmax is a new variant created by replacing nCas9 (H840A) in PE2 with nCas9 (R221K/N394K/H840A) to enhance the prime editing process (Zong et al., 2022).

Twin PE and GRAND editor were developed to facilitate the replacement or knock-in of large indels of a desired gene sequence. They use a pair of specially constructed pegRNAs with two RTs that are not homologous with the targeted sites but partly complement each other (Tang et al., 2020; Nelson et al., 2022). Prime editing offers several advantages over traditional HDR mechanisms and supports the replacement of small indels in specific target gene sequences and all kinds of base substitutions. It has great potential for precise genome editing and holds promise for various applications in genetic research and biotechnology. The prime editing technology is still in its early stages and faces a number of challenges before it can realize its potential. Its widespread use is also constrained by the large differences in editing efficiency between target loci and cell types. Base on, preliminary research in plants and other organisms, the efficacy of prime editing is influenced by a number of parameters, which include the original source of the RT enzyme, the thermos-stability and binding ability of the reverse transcriptase enzyme (RT) to its target site, the size of the RT template, the size of the PBS sequence, and the precise position of the nicking sgRNA in the unaltered strand (Anzalone et al., 2019; Lin et al., 2020; Tang, Sretenovic, Ren, Jia, Li, Fan, Yin, Xiang, Guo and Liu, 2020). Thermo-stability, the size of the RT template, and its capacity to bind to the target site are among the variables that made a noticeable impact on the editing effectiveness in both plant and human cells (Lin et al., 2020; Marzec et al., 2020). In addition to the PE’s efficiency, it is still challenging to successfully introduce prime-editing systems into the target cells. Editing efficiency is increased by up to 3.0 fold by mutations that boost RT’s thermos-stability and ability to bind to the target (Anzalone et al., 2019). Additionally, many RT from various sources had variable editing efficiency, as shown by Lin et al. (2020), which showed that RT produced by the Moloney murine leukemia virus (M-MLV) exhibited higher editing efficiency than RT obtained by the Cauliflower mosaic virus (CaMV). According to a recent study, the length of the RT template had a substantial impact on editing efficiency, particularly in plant cells, although the position and length of the PBS of nicking sgRNA did not significantly affect editing effectiveness (Lin et al., 2020; Tang et al., 2020).

### 5.6 CRASPASE: a new CRISPR-Cas tool in genetic engineering with protease activity

The CRISPR-Cas system have various types based on Cas effector composition, with Class 1 systems (Types I, III, and IV) comprising multi-subunit effector complexes and Class II systems (Types II, V, and VI) containing single-subunit protein effectors (Liu et al., 2022b). Bacteria have ubiquitous type III CRISPR-Cas immunity, generally mediated by a multi-subunit effector complex with various subtypes from type III-A to F (van Beljouw et al., 2021). In the type III CRISPR-Cas system, RNA-guided DNA/RNA degradation is not the only mechanism to confer immunity in prokaryotes; an alternate mechanism is RNA-guided secondary messenger production and signaling to induce immune response such as collateral RNA cleavage (Huang and Zhu, 2021; Rouillon et al., 2021; Steens et al., 2022; L. Wang et al., 2019). Type III-E is a newly identified aberrant type III CRISPR-Cas system that includes a gRAMP (giant repeat associated-mysterious protein), which is CRISPR RNA-guided RNA endonuclease that recognizes the target RNA sequence and cleaves single-stranded RNA at two specific positions six nucleotides apart (van Beljouw et al., 2021). Type III-E effector gRAMP/Cas7-11 combines with caspase-like peptidase TPR-CHAT (tetratricopeptide repeat-caspase HetF associated TPRs), which forms the CRASPASE (CRISPR-guided caspase complex) that mediates target RNA-influenced protease activity to acquire viral immunity (van Beljouw et al., 2021; Liu et al., 2022b; Cui et al., 2022; Hu et al., 2022; Yu et al., 2022). gRAMP/Cas7-11 comprises various type III domains, including four Cas7 domains and Cas11 fused as a single large protein called gRAMP encoded by a single gene, and forms a functional complex with TPR-CHAT (Hu et al., 2022). TPR-CHAT reportedly cleaves gasdermin in prokaryotes, a component involved in programmed cell death by membrane pore formation, resulting in cellular suicide (Johnson et al., 2022). Type III-E gRAMP/Cas7-11 exhibits target-specific RNA cleavage with no collateral activity and cytotoxicity, making it a promising tool for RNA knockdown and editing (Liu et al., 2022b; Cui et al., 2022; Ekundayo et al., 2022) Moreover, the type III-E system does not affect cell viability in mammalian cells (van Beljouw et al., 2021; Özcan et al., 2021; Liu et al., 2022b). Non-self-target RNA binding activates ssDNase and cyclic adenylate synthetase activities of Cas10 signature protein allosterically to destroy invading genetic material and switches off after the target RNA cleavage by csm3/cmr4 to prevent host damage by continuous enzymic activities (Kazlauskiene et al., 2016, 2017; Niewoehner et al., 2017; Rouillon et al., 2018)). Type III-E system lacks signature Cas10 protein; TPR-CHAT is present (Cui et al., 2022).

Recent studies have uncovered the structural characteristics and underlying mechanisms of gRAMP (giant repeat associated-mysterious protein) and CRASPASE (CRISPR-guided caspase complex) within the type III-E CRISPR-Cas system (van Beljouw et al., 2021; Özcan et al., 2021; Liu et al., 2022a, 2022b; Ekundayo et al., 2022; Hu et al., 2022; Yang and Patel, 2022). Liu et al. (2022b) reported the structure and mechanism of gRAMP complex with target RNA and non-target RNA, and the CRASPASE complex binds with invading target RNA to activate TPR-CHAT activity, which cleaves csx30 protein and activates programmed cell death as an antiviral immune response. Liu et al. (2022b) also showed the cryo-EM structures of type III-E CRISPR-Cas complex with target RNA and non-target RNA binding and subsequent TPR-CHAT activation to confer antiviral response. Their comparative structural analysis of the CRASPASE complex bound to CTR (cognate target RNA) and NTR showed that CTR binding induces conformational alterations in the TPR domain and activates TPR-CHAT protease activity. In contrast, NTR binding showed no conformational changes in TPR-CHAT. Structural analysis of CRASPASE and TR (target RNA) complex revealed that a 5′ tag of crRNA complementary to a 3′ anti-tag of TR is necessary for csx30 cleavage by TPR-CHAT and subsequent viral immunity (Liu et al., 2022b). The same study also demonstrated the CTR binding induces csx30 cleavage, which is bound to csx31 and SbRpoE. However, the specific functions of SbRpoE, csx30, and csx31 in the immune response of the type III-E CRISPR-Cas system remain unknown. Overall, this study identified the mechanistic role of gRAMP and TPR-CHAT (CRASPASE) in type III-E CRISPR-Cas immunity (Liu et al., 2022b). In other studies, the role of TPR-CHAT in Cas7-11-crRNA was unclear in *Scalindua brodae* (SbCas7-11) and *Desulfonema ishitimonii* (DiCas7-11). A study led by van Beljouw et al. (2021) reported that in SbCas7-11 TPR-CHAT does not affect Cas7-11 nuclease activity, while the similar study by Özcan et al. (2021) showed DiCas7-11 regulated Cas7-11 nuclease activity induced by TPR-CHAT. *In vitro* assays revealed that DmTPR-CHAT can rigidly obstruct DmCas7-11 nuclease activity by shortening target RNA binding. Structural and biochemical analysis of DmTPR-CHAT and DmCas7-11 unveiled the regulation of Cas7-11 by TPR-CHAT by stabilizing interactions between the NTD (N-terminal domain) of TPR-CHAT and the Cas11-like domain and insertion finger domain of Cas7-11, and CLD transiently inhibited target RNA binding and disturbed Cas7-11 nuclease activity (Ekundayo et al., 2022). The insertion finger and NTD involvement in the CRASPASE complex indicate a way forward in engineering this system to regulate Cas7-11 activity (Liu et al., 2022a; Ekundayo et al., 2022). Cryo-EM structures of Cas7-11 complexed with target RNA were used to determine the molecular basis of crRNA processing and target RNA cleavage by DiCas7-11, revealing programmable RNA cleavage in *in vitro* and mammalian cells (Niwa et al., 2020; Catchpole and Terns, 2021; van Beljouw et al., 2021; Özcan et al., 2021; Goswami et al., 2022).

**FIGURE 6:**
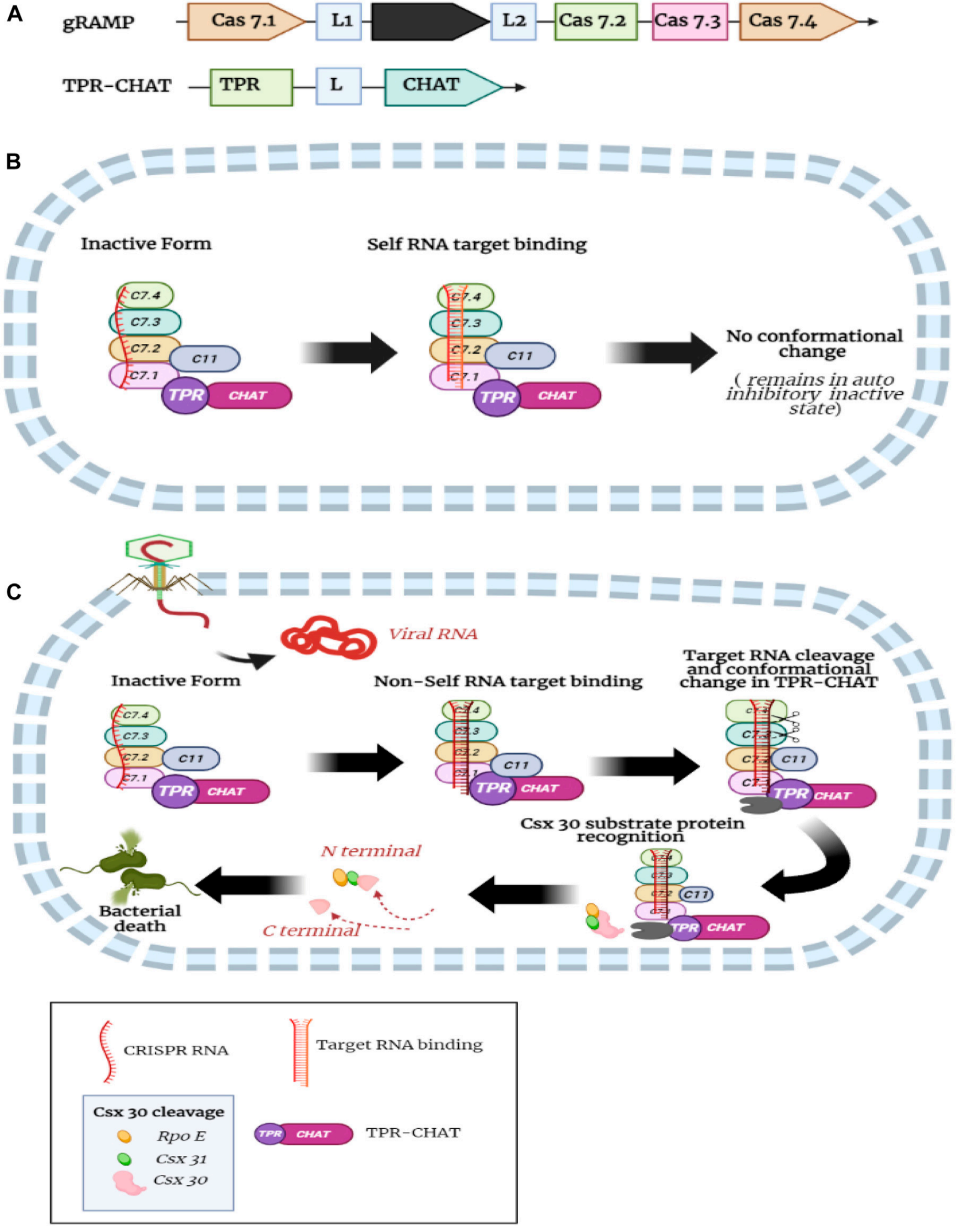
**(A)** Domain organization of gRAMP (Black box represent cas11-like domain) and TPR-CHAT protein; **(B)** When CRASPASE complex bind to self RNA target, the 3′ anti-tag sequences follow the distinctive path from the non-self target RNA 3′ flanking sequence; however, it induces less conformational alterations in TPR-CHAT and stays in an auto-inhibitory state. **(C)** when CRASPASE complex bind to non-self RNA target, Soon after, the non-self target RNA binding and 3′ flanking sequence of non-self target RNA incite conformational alterations in the linker domain of TPR-CHAT, results in activation of the protease activity of CHAT, and cleaving csx30 protein substrate and causes cell death (Created with BioRender.com).

A recent study identified the structural basis of self and non-self RNA target recognition and TPR-CHAT protease activity regulation in the type III-E gRAMP-TPR-CHAT CRISPR-Cas system (Cui et al., 2022). Moreover, the study uncovered the structural basis of self (host) RNA and non-self RNA binding to the gRAMP-crRNA complex (Cui et al., 2022). TPR-CHAT forms complex with gRAMP and stays in an auto-inhibitory inactive state. Soon after, the non-self target RNA binding and 3′ flanking sequence of non-self target RNA incite conformational alterations in the linker domain of TPR-CHAT, resulting in reshuffling in the catalytic pocket of CHAT protease, activating the protease activity of CHAT, and cleaving csx30 protein substrate. Following cleavage of the RNA targets by csm3 domain releases target RNA turning off the protease activity of TPR-CHAT (Figure 6C). In contrast, binding the target self RNA b, the 3′ anti-tag sequences follow the distinctive path from the non-self target RNA 3′ flanking sequence; however, it induces less conformational alterations in TPR-CHAT and stays in an auto-inhibitory state (Figure 6B). The base-pairing capabilities of the 5′ repeat tag of crRNA and 3′ sequence of the RNA targets at different positions are important for differentiating non-self and self RNA. Subsequently, the nuclease activity of the csm3 domains primarily controls TPR-CHAT protease activity to host cell damage (Cui et al., 2022).

The collective research on CRASPASE mechanism opens up possibilities for developing a nuclease-protease-based CRISPR-Cas system for the precise manipulation of RNA without causing collateral activity and cell toxicity (Liu et al., 2022b; Cui et al., 2022; Ekundayo et al., 2022; Goswami et al., 2022; Yang and Pytatel, 2022). The exact mechanism of csx30 in bacterial cell death after cleavage by TPR-CHAT remains elusive, but the similarities with eukaryotic separase suggest interesting avenues for future research (Cui et al., 2022; Ekundayo et al., 2022; Hu et al., 2022). The recent discovery that the binding and cleavage of Csx30 regulates the transcriptional activity of RpoE suggests that transcriptional activity might be involved in spacer acquisitions and distinct immune responses (Strecker et al., 2022). Csx30, cleaved into C-terminal and N-terminal fragments by protease activity of TPR-CHAT, switches off after RNA cleavage by gRAMP, resulting in the timely control of the immune response and binding to the new target RNA (Liu et al., 2022b; Hu et al., 2022; Yang and Patel, 2022). Structural and biochemical analysis revealed that the gRAMP/Cas7-11 cleaves target single-stranded RNA at cas7.2 and Cas7.3 active sites before the fourth and 10th nucleotide positions, respectively (van Beljouw et al., 2021; Goswami et al., 2022; Kato et al., 2022). Comparison between all the techniques used for gene editing including, ZFN, TALEN, CRISPR-Cas, base editors, and prime editors is shown in (Table 3).

**TABLE 3:** Comparison between ZFN, TALEN, CRISPR-Cas, base editors, and prime editors.

ZFN	TALEN	CRISPR-Cas	Base editors	Prime editors
Protein-based genome editing tool comprising DNA cleavage domains *fok1* and synthetic zinc finger-based DNA-binding domain	Nuclease-dependent genetic engineering tool that contains the nuclease domain of the *fok1* enzyme and TALE protein having customizable DNA-binding repeats	RNA-based genome editing method comprising crRNA, trans-activating CRISPR RNA (tracr RNA), and cas9 enzyme	Newly developed genome engineering tool, classified into C-to-G base editors (CGBEs), cytosine base editors (CBEs), and adenine base editors (ABE), all of which have different compositions	RNA-dependent genome engineering method comprising nCas9 (H840A) and the Moloney murine leukemia virus reverse transcriptase (M-MLV-RT)
Endonuclease				
*fok1*	*fok1*	Cas9	DCas	pegRNA
NA	NA	Cas protein: Cas9	Cas protein: nCas9 (D10A)	Cas protein: nCas9(H840A)
NA	NA	RNA: single guide RNA (sgRNA)	RNA: sgRNA	RNA: prime editing guide RNA (PegRNA)
NA	NA	Reverse transcriptase: No	Reverse transcriptase: No	Reverse transcriptase: Yes
Mutation types				
Gene disruption and insertions	Gene disruption and insertions	All types of base insertions, deletions, and substitutions	Transition mutations (no insertion and deletions)	All types of base insertions, deletions, and substitutions
			(CBE) for C:G to T:A transition	
			(ABE) for A:T to G:C transition	
			(CGBEs) for inducing transversion of C:G into G:C	
Origin				
Artificial gene editing technique	Artificial gene editing technique	Naturally occurring RNA-based bacterial defense mechanism	Engineered nucleases	Engineered nucleases
Mutation rate				
Moderate	Moderate	Low	High	Very high
Target recognition efficiency				
High	High	High	Very high	Very high
Off-target effects				
High	Low	Variable	Very low	Very low

## 6 Regulation of CRISPR edited crops

Gene editing tools such as CRISPR-Cas system has revolutionized the field of agriculture by maximizing the development of novel crop varieties with improved traits. CRISPR-Cas approach does not fit the definition of genetically modified organisms (GMO) as it does not involves transferring the foreign gene (free of transgenes) into the mutated plant. Nevertheless, the potential of this precision gene editing technology in future relies on the establishment of a clear and internationally recognized regulatory system for these CRISPR edited crops. CRISPR edited crops can produce products that shows resemblance to those produced by traditional breeding techniques, the only difference lies in terms of its regulatory systems adopted across different countries. In general, there are two regulatory approaches adopted by different countries - product based regulatory approach and process based regulatory approach. Compared to product based, process based regulatory approach is a time consuming and expensive regulatory approach (Ahmad, Ghouri, et al., 2021; Ahmad, Munawar, et al., 2021; Gupta et al., 2021).

### 6.1 Product based regulatory approach

Product based regulatory approach involves assessment of the health and environment risk based on the final product rather than accessing the process involved in generating the final product (Sprink et al., 2016).

Product based approach is based on the assessment on final product leading to the possibility of two conditions. First condition, if transgene is present then GMO regulations will be applied. In second condition, if transgene is absent then the product can be commercialized in the market without following GMO regulations. Canada is one such country that follows this regulatory system, which involves the evaluation of any “novel trait” in plants for potential risks. The novel trait inserted must be unique to the environment, have implications for the plant’s usage, and should be associated with health or environmental safety concerns. Other countries like Argentina and US embraced same approach for risk assessment and regulation of genetically edited crops. In conclusion, this approach is time saving that provides rapid benefits to the society.

### 6.2 Process based regulatory approach

Process based regulatory approach involves assessment of risk analysis based on reviewing the procedure involved in generating the final product rather than accessing the final product. The European Union, Court of Justice (ECJ) in 2018 ordered that all organisms modified through genome editing must be classified as genetically modified organisms (GMOs). As a result, these GMOs are subjected to significant regulatory burdens under the EU GMO Directive. In contrast, other techniques such as chemical and radiation-induced mutagenesis are exempted from these EU GMO Directive because they have a long history of safe use (Friedrichs et al., 2019). New Zealand follows similar GM biosafety regulations foe genome editing techniques applying the similar rules to crops modified through genetic engineering. However, these regulations have posed challenges to innovation in the field of plant breeding. Countries like US have adopted different versions of process based regulatory approach, where they regulates GMOs by assessing the risks of novel traits or products of traditional varieties (Ishii and Araki, 2017). In Argentina, if genome-edited plants do not exhibit genetically modified traits such as antibiotic resistance or herbicide tolerance, they are not subjected to specific regulatory requirements. Genome edited products that lack transgenes are exempted from GMO regulation in Argentina, which has also embraced process-based regulatory approach. In conclusion, Process based regulatory approach in adopted by countries like India and Norwegian. On the other hand countries such as US regulates in a combined approach both process and product based regulatory approach.

## 7 Conclusion and future prospects

Genome editing techniques, particularly CRISPR-Cas, have revolutionized the field of plant breeding and agriculture, offering unprecedented precision and efficiency in modifying target genes. While ZFN and TALEN were early pioneers in gene editing, their complexity and limitations made them less practical for widespread use in plants. In contrast, CRISPR-Cas9 has emerged as a more accessible and versatile tool, driving significant advances in crop improvement.

CRISPR-Cas has been used successfully to enhance various agricultural traits, such as nutritional content, pest and herbicide resistance, yield, and stress tolerance. As our understanding of the CRISPR-Cas system expands and Cas variants evolve, base editors and prime editors hold promise in further refining plant genome engineering. For example, the more recently identified CRASPASE complex, which can successfully cleave proteins, could be used for genome and protein editing in organisms. Recently, a new class of RNA-guided system in prokaryote is identified termed as OMEGA (Obligate Mobile Element-guided activity) which comprises RNA-guided endonuclease protein TnpB (Transposase B) and the transposon end region transcribed non-coding RNA (ncRNA or ωRNA). OMEGA system is an ancestor of CRISPR-Cas system and TnpB share structural and functional similarity with Cas12 protein. Fanzor (Fz) protein have been identified in eukaryotes which is an eukaryotic RNA-guided endonucleases, they share close homology with TnpB. These findings suggest that Fanzor could be eukaryotic CRISPR-Cas or OMEGA system. Fanzor from eukaryotes and OMEGA system from prokaryotes have been employed for genome editing in mammalian cells. These editing tools offer new opportunities to achieve precise modifications, reducing unwanted off-target effects and increasing gene editing efficiency.

The promising future of genome editing in agriculture will likely result in the commercialization of an increasing number of genetically engineered crops. However, with the potential benefits come ethical and safety considerations. Careful assessment of the potential risks and impacts on human health, the environment, and non-target organisms is crucial as we transition from laboratory research to field applications. Measures to minimize undesirable consequences should be recorded and publicly acknowledged because avoidable off-target effects may occur. All successfully produced transgenic foods and crops must be registered, and market management must be standardized. It is also essential to stay vigilant about the potential cytotoxic effects of the components used in genome editing methods (CRISPR-Cas, TALEN, and ZFN). Continuous research and risk assessment will be crucial in advancing the field responsibly and sustainably.
